# Characterization of the Zebrafish Cell Landscape at Single-Cell Resolution

**DOI:** 10.3389/fcell.2021.743421

**Published:** 2021-10-01

**Authors:** Mengmeng Jiang, Yanyu Xiao, Weigao E, Lifeng Ma, Jingjing Wang, Haide Chen, Ce Gao, Yuan Liao, Qile Guo, Jinrong Peng, Xiaoping Han, Guoji Guo

**Affiliations:** ^1^Bone Marrow Transplantation Center, The First Affiliated Hospital, Zhejiang University School of Medicine, Hangzhou, China; ^2^Liangzhu Laboratory, Zhejiang University Medical Center, Hangzhou, China; ^3^Center for Stem Cell and Regenerative Medicine, Zhejiang University School of Medicine, Hangzhou, China; ^4^College of Animal Sciences, Zhejiang University, Hangzhou, China; ^5^ZJU-UOE Institute, Zhejiang University School of Medicine, Haining, China

**Keywords:** zebrafish, single cell, sequencing, cross-species analysis, regeneration

## Abstract

Zebrafish have been found to be a premier model organism in biological and regeneration research. However, the comprehensive cell compositions and molecular dynamics during tissue regeneration in zebrafish remain poorly understood. Here, we utilized Microwell-seq to analyze more than 250,000 single cells covering major zebrafish cell types and constructed a systematic zebrafish cell landscape. We revealed single-cell compositions for 18 zebrafish tissue types covering both embryo and adult stages. Single-cell mapping of caudal fin regeneration revealed a unique characteristic of blastema population and key genetic regulation involved in zebrafish tissue repair. Overall, our single-cell datasets demonstrate the utility of zebrafish cell landscape resources in various fields of biological research.

## Introduction

The zebrafish (*Danio rerio*) is widely accepted as an *in vivo* model to study vertebrate biological processes for its similarity with human (Torraca and Mostowy, 2018). The combination of optical accessibility and genetic tractability make zebrafish an important system to investigate gene regulation and cell lineage specification. Recently, owing to the advent of high-throughput single-cell RNA sequencing (scRNA-seq), it is now possible to fully define the cell-type composition in complex biological systems (Klein et al., 2015; Macosko et al., 2015). Single-cell data resources are important for understanding cellular regulations and functions (Gupta et al., 2018). The mapping of cell atlases for whole organisms such as human, mouse, *Caenorhabditis elegans* larvae, planarians, and cnidarians has been achieved (Fincher et al., 2018; Han et al., 2018; Plass et al., 2018; Sebe-Pedros et al., 2018; Packer et al., 2019; Han et al., 2020). Cellular heterogeneity and developmental trajectory in zebrafish embryos have also been analyzed (Tang et al., 2017; Alemany et al., 2018; Farrell et al., 2018; Spanjaard et al., 2018; Wagner et al., 2018; Farnsworth et al., 2019). However, a comprehensive adult zebrafish cell landscape and the related molecular networks have not been fully characterized.

As a powerful model, zebrafish are able to fully regenerate many tissues, such as the brain, spinal cord, kidney, heart, liver, and caudal fin (Tanaka, 2016). It remains unclear why mammals usually have limited tissue repair capacity, while lower vertebrates possess stronger ability to regenerate. Powerful molecular tools have increased our understanding of regenerative mechanisms, such as lineage tracing with transgenic lines, live imaging, and scRNA-seq (Lin et al., 2021). Profiling of *Xenopus* tail regeneration revealed a previously unrecognized cell type at single-cell resolution, which was associated with key regenerative pathways (Aztekin et al., 2019). Single-cell analysis of axolotl limb regeneration showed the characteristic and molecular dynamics of blastema cells (Gerber et al., 2018). Zebrafish caudal fin regeneration is also a good system to explore the process of blastema cell formation. However, the unique signatures of blastema cells and the underlying molecular mechanisms during caudal fin regeneration remain unclear.

Here, we used Microwell-seq to construct an initial compendium of a “Zebrafish Cell Landscape,” comprising more than 250,000 cells isolated from zebrafish embryo and adult tissues. Single-cell analysis of caudal fin regeneration displayed a unique characteristic of blastema population and key signaling pathways involved in zebrafish tissue repair. Our results provide a general and valuable resource for future studies on zebrafish.

## Materials and Methods

### Zebrafish Husbandry, Fin Amputation, and BMP Inhibitors Treatment

Zebrafish (*D. rerio*) wild-type Tübingen strain was raised and maintained in standard zebrafish units at Core Facilities, Zhejiang University School of Medicine. The adult zebrafish aged from 4 to 12 months were studied. The zebrafish research analysis conducted in this study was approved by the Ethics Committee of the Zhejiang University Laboratory Animal Center.

After caudal fins were amputated one or two segments proximal to the origin of bifurcation, adult zebrafish (6 months old) were kept in system water at 28.5°C and the system water with BMP inhibitors was replaced daily. 10 μM DMH1 (Target Mol), and 10 μM Dorsomorphin (Target Mol) were used as specific BMP inhibitors. All were dissolved in DMSO and final DMSO concentration in fish water was 0.1%. The control zebrafish were kept in fish water with 0.1% DMSO. The lengths of caudal fin were detected after 3 days of BMP inhibitors treatment.

### Fabrication of Microwell Device

The diameter and depth of the microwells are 28 and 35 μm, respectively. First, a silicon plate containing 100,000 microwells was manufactured by Suzhou Research Materials Microtech Co. Ltd. (Suzhou, China). The silicon microwell plate was then used as a mold to make a PDMS plate with the same number of micropillars. Prior to experiments, a disposable agarose microwell plate was made by pouring 5% agarose solution onto the surface of the PDMS plate. Both the silicon and the PDMS plates are reusable. One silicon microwell plate allows almost permanent use.

### Synthesis of Barcoded Beads

Magnetic beads (diameter 20–25 μm) coated with carboxyl groups were provided by Suzhou Knowledge & Benefit Sphere Tech. Co., Ltd. (Suzhou, China^1^). The barcoded oligonucleotides on the surfaces of the beads were synthesized by three rounds of split-pool. All the sequences used are the same as those reported previously (Han et al., 2018).

For each batch of bead synthesis, 300–350 μl of carboxyl magnetic beads (50 mg/ml) was washed twice with 0.1 M MES (2-[N-morpholino]ethanesulfonic acid). The beads were then suspended in a final volume of 635 μl 0.1 M MES. EDC [1-ethyl-3-(3-dimethylaminopropyl) carbodiimide hydrochloride] (3.08 mg) was added to the beads, and 6.2 μl of beads was then placed in each well of a 96-well plate. Amino-modified oligonucleotides (2.5 μl, 50 μM in 0.1 M MES) were then added to each well. After vortexing the mixture and incubating it for 20 min at ambient temperature, 0.5 μl of mix (6 mg of EDC in 100 μl of 0.1 M MES) was distributed into each well. After an additional round of vortexing and incubation for 20 min at ambient temperature, an additional 0.5 μl of mix (6 mg of EDC in 100 μl of 0.1 M MES) was distributed into each well. After vortexing and incubation for 80 min at ambient temperature, the beads were collected in 1 ml of 0.1 M PBS containing 0.02% Tween-20. After centrifugation, the supernatant was carefully removed. The beads were then washed twice in 1 ml of TE (pH 8.0).

In the second split-pool, the beads were washed with water and divided among the wells of another 96-well plate containing PCR mix (1 × Phanta Master Mix, Vazyme) and 5 μM oligonucleotides. The oligonucleotides in each tube encoded a sequence with reverse complementarity to linker 1, a unique barcode and a linker 2 sequence. The PCR program was as follows: 94°C for 5 min; 5 cycles of 94°C for 15 s, 48.8°C for 4 min, and 72°C for 4 min; and a 4°C hold. The third split-pool procedure was the same as the second one. The PCR program was as follows: 94°C for 5 min, 48.8°C for 20 min, 72°C for 4 min and a 4°C hold. Beads were mixed sufficiently between denaturation (95°C) and primer annealing (48.8°C) in every cycle. The oligonucleotides used in each tube encoded a linker 2 reverse-complementary sequence, a unique barcode, a unique molecular identifier (UMI) sequence and a poly-T tail. All the oligonucleotides were synthesized by Sangon Biotech Co. Ltd. with HPLC purification. To remove the chains without the third barcoded sequence, beads were collected and suspended in 200 μl of exonuclease I mix [containing 1× exonuclease I buffer and 1 U/μl exonuclease I (NEB)], and incubated at 37°C for 15 min (beads were mixed by rotary mixer). After being washed with 200 μl of TE-TW and 200 μl of 10 mM Tris-HCl pH 8.0, beads were resuspended in 1 ml of ddH_2_O. To remove complementary chains, the beads were placed in a 95°C water bath for 6 min and separated using a magnet, removing the supernatant quickly, 2 times. The beads could be stored in TE-TW (10 mM Tris pH 8.0, 1 mM EDTA, 0.01% Tween-20) for 4 weeks at 4°C.

### Cell Preparation

Dissociation of embryonic tissues was performed similarly as previously described (Manoli and Driever, 2012). In brief, 50–100 zebrafish embryos were grown to the indicated times and chorions were removed by incubating in 1mg/ml Pronase (Sigma) for 3–4 min followed by washing in 0.5× Danieau Buffer. [10× Danieau Buffer = 174 mM NaCl, 2.1 mM KCl, 1.2 mM MgSO4, 1.8 mM Ca(NO3)2, 15 mM HEPES, pH 7.6]. Yolk were removed by blowing in deyolking buffer [55 mM NaCl, 1.8 mM KCl, 1.25 mM NaHCO3] for 10 times followed by washing in 0.5× Danieau Buffer. Embryo tissues were triturated to homogeneity in 1–5 ml FACSmax cell dissociation solution (AMS Biotechnology) and incubated for 4–5 min at room temperature. Then embryonic single cells were collected after passage through a 40-μm strainer (Biologix). Zebrafish tissues were carefully dissected, put to cold DPBS and minced into ∼1-mm pieces on ice using scissors. The tissue pieces were transferred to a 15-ml centrifuge tube, rinsed twice with cold DPBS and suspended in 5 ml of a solution containing dissociation enzymes. The samples were treated with various enzymes at 37°C for different amounts of time (Supplementary Table 1). During the dissociation, the tissue pieces were pipetted up and down gently several times until no tissue fragments were visible. The dissociated cells were centrifuged at 300 × *g* for 5 min at 4°C and then re-suspended in 3 ml of cold DPBS. After passage through a 40-μm strainer (Biologix), the cells were washed twice, centrifuged at 300 × *g* for 5 min at 4°C, and re-suspended at a density of 1 × 10^5^ cells/ml in cold DPBS containing 2 mM EDTA.

### Cell Collection and Lysis

Cell concentration should be carefully controlled during Microwell-seq. Both cell and bead concentrations were estimated using a haemocytometer. The proper cell concentration is ∼100,000/ml (with 10% of the wells occupied by single cells). The proper bead concentration is ∼1,000,000/ml (with every well occupied by single beads). An evenly distributed cell suspension was pipetted onto the microwell array, and extra cells were washed away. To eliminate cell doublets, the plate was inspected under a microscope. Cell doublets were reduced by pipetting over the region of high cell density. The bead suspension was then loaded into the microwell plate, and the plate was placed on a magnet. Excess beads were washed away slowly. Cold lysis buffer (0.1 M Tris-HCl pH 7.5, 0.5 M LiCl, 1% SDS, 10 mM EDTA, and 5 mM dithiothreitol) was pipetted over the surface of the plate and removed after 12 min of incubation. The beads were then collected, transferred to an RNase-free tube, and washed once with 1 ml of 6 × SSC, once with 500 μl of 6 × SSC and once with 200 μl of 50 mM Tris-HCl pH 8.0. Finally, ∼50,000 beads were collected in a 1.5-ml tube.

### Reverse Transcription

In this procedure, the instructions from the Smart-seq2 protocol were followed (Picelli et al., 2014). Briefly, 20 μl of RT mix was added to the collected beads. The RT mix contained 200 U SuperScript II reverse transcriptase, 1× Superscript II first-strand buffer (Takara), 20 U RNase Inhibitor (Sangon), 1 M betaine (Sigma), 6 mM MgCl_2_ (Ambion), 2.5 mM dithiothreitol, 1 mM dNTP and 1 μM TSO primer. The beads were incubated at 42°C for 90 min with mixing on a rotary mixer and then washed with 200 μl of TE-SDS (1 × TE + 0.5% sodium dodecyl sulfate) to inactivate reverse transcriptase.

### Exonuclease I Treatment

The beads were washed with 200 μl of TE-TW and 200 μl of 10 mM Tris-HCl pH 8.0, resuspended in 100 μl of exonuclease I mix containing 1× exonuclease I buffer and 1 U/μl exonuclease I (NEB), and incubated at 37°C for 60 min with mixing on a rotary mixer to remove oligonucleotides that did not capture mRNA. The beads were then pooled and washed once with TE-SDS, once with 1 ml of TE-TW and once with 200 μl of 10 mM Tris-HCl pH 8.0.

### cDNA Amplification

The beads were distributed into 4 PCR tubes. To each tube, 12.5 μl of PCR mix [1 × HiFi HotStart Readymix (Kapa Biosystems) and 0.1 μM TSO_PCR primer] was added. The PCR program was as follows: 98°C for 3 min; 4 cycles of 98°C for 20 s, 65°C for 45 s, and 72°C for 6 min; 10–14 cycles of 98°C for 20 s, 67°C for 20 s, and 72°C for 6 min; 72°C for 10 min; and a 4°C hold. After pooling all PCR products, AMPure XP beads (Beckman Coulter) were used to purify the cDNA library.

### Transposase Fragmentation and Selective PCR

The purified cDNA library was fragmented using a customized transposase that carries two identical insertion sequences. The customized transposase was included in the TruePrep DNA Library Prep Kit V2 for Illumina (Vazyme). The fragmentation reaction was performed according to the instructions provided by the manufacturer. We replaced the index 2 primers (N5××) in the kit with our P5 primer to specifically amplify fragments that contain the 3′ ends of transcripts. Other fragments will form self-loops, impeding their binding to PCR primers. The PCR program was as follows: 72°C for 3 min; 98°C for 30 s; 5 cycles of 98°C for 15 s, 60°C for 30 s, and 72°C for 3 min; 72°C for 5 min; and a 4°C hold. The PCR product was purified using AMPure XP beads. Then, 25 μl of PCR mix (1× HiFi HotStart Readymix and 0.1 μM 2100 primer) was added to each sample. The PCR program was as follows: 95°C for 3 min; 8 cycles of 98°C for 20 s, 60°C for 15 s, and 72°C for 15 s; 72°C for 5 min; and a 4°C hold. To eliminate primer dimers and large fragments, AMPure XP beads were then used to purify the cDNA library. The size distribution of the products was analyzed on an Agilent 2100 bio-analyzer, and a peak in the 400–700 bp range was observed. Finally, the samples were subjected to sequencing on an Illumina Hiseq system.

### Whole Mount *in situ* Hybridization

Whole mount *in situ* hybridization (WISH) with DIG (Digoxyigenin)-labeled was performed using standard protocols as described previously (Thisse and Thisse, 2008). To resolve the problem of high background in caudal fin tissues, sense probes were also performed for control. Sequences were listed as follows: *and1* (antisense probe: CGGCGCAGGCGGCAG; sense probe: CCAGAAAGCCCCCTCT), *c1qtnf5* (antisense probe: AGGAA GCCACGGTGT; sense probe: GCAGACATGGCCTCT), *clu* (antisense probe: CTGAAAGAAGCCGT; sense probe: GACCA CATGCAGATG), *ecrg4a* (antisense probe: CAGAGCAGAGAA TCAT; sense probe: GACAGCACTGTCTCT), *fgfbp2a* (antisense probe: GACGAAGGAGCATGC; sense probe: CCGAGCAGCTC TCGC). Photos were taken under a Leica M216 optics.

### Processing of the Microwell-Seq Data

Standard procedures for processing the Microwell-seq datasets were performed using the protocols described in the previously published paper (Han et al., 2018). Reads from zebrafish cell landscape data were aligned to the *D. rerio* GRCz10 genome using STAR (Dobin et al., 2012) and the digital gene expression (DGE) data matrixes were obtained using the Drop-seq Core Computational Protocol^2^ with default parameters. For quality control, we filtered out cells with detection of less than 500 transcripts. Cells with high proportion (>20%) of transcript counts derived from mitochondria-encoded genes were also excluded.

### Clustering of Single Cell Data Matrix

Seurat (V3.0) (Satija et al., 2015) was used to perform clustering analysis of single cell data from different tissues. The DGE data was used as inputs. Cells from the pre-processed data and genes expressed in more than 3 cells were selected for further analysis. The filtered data was log_2_(TPM/100 + 1) transformed, then the number of UMI and the percentage of mitochondrial gene content were regressed out according to published method (Buettner et al., 2015). About 2,000 various genes with average expression more than 0.5 and a dispersion greater than 0.125 were used as inputs for initial principal component analysis (PCA) and number of principal components (PCs) used for Non-linear Dimensional Reduction (t-SNE) analysis is chosen according to the PCElbowPlot function and JackStrawPlot function. For clustering, we set different resolution parameters between 0.6 and 4 in FindAllCluster function and narrowed down to certain cluster numbers by distinguishing differential genes among clusters. The heatmap, produced by DoHeatmap function is one of basis for judging the quality of clustering. These parameters, including resolution and number of principal components, were adjusted on per-tissue basis. The default wilcox rank sum test was used by running FindAllMarkers function in Seurat to find DEGs in each cluster. Gene ontology (GO) analysis of DEGs was performed with ToppGene^3^. Finally, we annotate each cell type by extensive literature reading and searching for the specific gene expression pattern.

For processing of the complete zebrafish tissue dataset (258,902 cells), we used Scanpy (Wolf et al., 2018) in python environment to perform the analysis. Background-removed DGE data with cells analyzed in each tissue and genes expressed in at least 20 cells was the used as inputs for Scanpy. Then the DGE data was log_2_(TPM/10 + 1) transformed. We selected about 3,000 highly-variable genes according to their average expression and dispersion, regressed out UMI and gene numbers, scaled each gene to unit variance with clip values exceeding a standard deviation of 10. We chose about 50 PCs for the PCA, and we computed the neighborhood graph of cells. We then used the Louvain clustering to cluster cells with resolution = 2.5 and *k* = 10. Finally, 63 clusters for the zebrafish landscape were produced and marker genes were calculated by Wilcoxon rank sum test.

### Cross-Species Transcriptome Comparison

To make the gene expression profiles of cross-species cell types comparable, we downloaded the homology correspondences between zebrafish and human, mouse, axolotl provided by dmodENCODE (Celniker et al., 2009). The gene expression profiles for zebrafish (this study) and other species (Gerber et al., 2018; Han et al., 2018, 2020) were normalized to the total number of transcripts and multiplied by 100,000. To attenuate the effects of noise and outliers, we used pseudo-cells (Tosches et al., 2018) for further analysis; each pseudo-cell was an average of 20 cells randomly selected from the same cell type. To compare cross-species transcriptomes, we performed MetaNeighbor (Crow et al., 2018) analysis between zebrafish and mouse. MetaNeighbor was formalized through neighbor voting based on cell–cell similarities. The mean area under the receiver operator characteristic curve (AUROC) scores was used to measure the similarity of cell types, and we chose 0.8 as the threshold. The Circlize package was used to view in the similarity of cell types among different species (Gu et al., 2014). The network was visualized using Cytoscape with the “edge-weighted spring embedded” layout (Shannon et al., 2003).

### Weighted Correlation Network Analysis

WGCNA (V1.69) was performed with default parameters (Langfelder and Horvath, 2008). In brief, high variable genes among the caudal fin regeneration single cells of interest were calculated using the distance to the median metric. An adjacency matrix, representing a “unsigned” gene network, was built setting the soft power parameter to 4 (calculated from the pick Soft Threshold function). Modules were identified using the function block wise Modules, with a minimum module size of 30 and the save TOMs parameter set to TRUE. The TOM dissimilarity measure (1- TOM) to the fourth power was then used to cluster genes with the function TOM plot. And the correlation of modules and gene expressed in each cell type was calculated by Pearson correlation coefficient.

### Virtual Inference of Protein-Activity by Enriched Regulon Analysis

VIPER (V1.20.0) was performed to computational inference of TF activity from gene expression profile data with default parameters (Alvarez et al., 2016). Briefly, an appropriate cell context-specific regulatory network was generated with ARACNE algorithm^4^ from caudal fin regeneration and non-regeneration single-cell gene expression profiles (Margolin et al., 2006). We then performed master regulator analysis and estimated its significance, including *P*-value and normalized enrichment score (NES), by comparing each regulon enrichment score to a null model using msVIPER algorithm^5^. Master TFs and cofactors regulated each other and their downstream target genes were visualized by Cytoscape (V3.8.2).

### Gene Set Enrichment Analysis

Signature enrichment of regeneration relevant clusters against others was performed by gene set enrichment analysis (GSEA) (V1.0). The GSEA was implemented using JAVA downloaded from the Broad Institute^6^. *D. rerio* pathway database for enrichment was downloaded from https://www.wikipathways.org/index.php/Download_Pathways.

### Receptor–Ligand Pairing Analysis

Analysis of potential receptor–ligand pairings was performed using the method CellPhoneDB (V1.0.0) (Vento-Tormo et al., 2018). First, we aggregated the gene expression levels of 20 cells from each cluster in the caudal fin regeneration. To eliminate the effect of variable cell numbers in each cluster, we randomly sampled three pseudo-cells for analysis. Only receptors and ligands expressed in more than 10% of the cells in the specific cluster were considered. By permuting cluster labels randomly 1,000 times to calculate the mean expression values of ligands and receptors, interaction was constructed as a receptor–ligand pairing matrix. Then, we used pairwise comparisons between all cell types and obtained a likelihood of P value to filter the false-positive interaction. The cut off was set with the mean expression greater than 0.1 and P values smaller than 0.1. We used the sum of the number of receptor–ligand pairs in each cell–cell pairing to indicate the strength of the cell–cell interactions.

### Statistical Analysis

All the results were presented as mean ± SEM. Two-tailed Student’s *t*-test was performed to compare the differences between two groups. All quantitative experiments were repeated at least 3 times independently.

## Results

### Constructing a Zebrafish Cell Landscape Using Microwell-Seq

**FIGURE 1:**
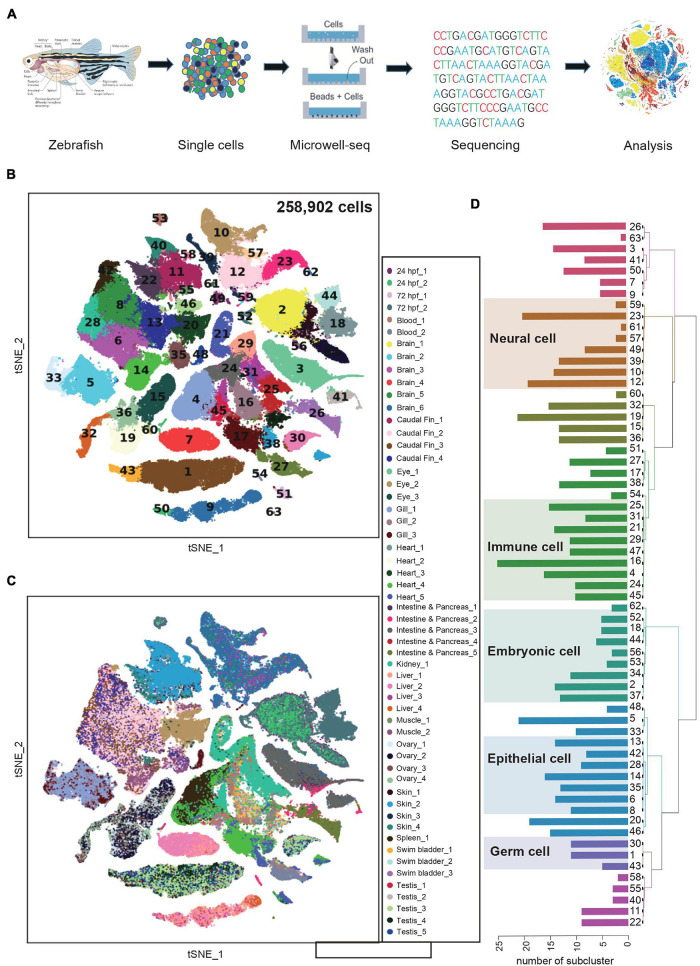
Constructing a zebrafish cell landscape using microwell-seq. **(A)** A schematic of the basic workflow for zebrafish cell landscape. Zebrafish tissues were analyzed. After dissociation, single cells were captured in single microwells. Cells were lysed, transcriptomes amplified and sequenced, reads mapped, and data analyzed. **(B)** t-distributed stochastic neighbor embedding (t-SNE) analysis of >250,000 single cells sampled from zebrafish embryo and adult tissues. In the t-SNE map, 63 main cell type clusters are labeled by different colors. Cell cluster markers are listed in Supplementary Table 1. **(C)** t-SNE analysis of >250,000 single cells sampled from zebrafish tissues. Tissue types and batches are labeled by different colors in the t-SNE map. hpf, hours post-fertilization. **(D)** Dendrogram showing relationships among 63 cell types. The bar chart on the left represents the number of sub-clusters contained in each main cluster. A total of 633 sub-clusters were obtained from 63 main clusters.

Single-cell RNA sequencing technologies enable identification of cell identity. To comprehensively characterize different cell types in zebrafish, we performed mRNA-seq expression profiling in individual cells isolated from major tissues using Microwell-seq (Han et al., 2018) (Figure 1A). We collected the pharyngula stage (24 h post-fertilization, hpf) and hatching stage (72 hpf) zebrafish embryos, as well as blood, brain, caudal fin, eye, gill, heart, intestine, kidney, liver, muscle, ovary, pancreas, skin, spleen, swim bladder, and testis samples from adult zebrafish. Tissues were obtained, carefully dissected and prepared into single-cell suspension (Supplementary Table 1). Single cells were then processed with single-cell mRNA-seq.

The single-cell transcriptomics data were processed by published pipelines (Macosko et al., 2015; Satija et al., 2015). In general, we analyzed >250,000 single cells with an average 537 genes and ∼1,180 UMIs per cell from zebrafish embryos and 16 adult tissues (Supplementary Figure 1A). To detect relationships between cells from different organs, we visualized all cells with t-distributed stochastic neighbor embedding (t-SNE) and grouped them with unbiased, graph-based clustering (Figure 1B). In a global view, we identified tissue cell types in 63 major clusters (Supplementary Table 1). Experimental batches from the same tissues were well controlled (Figure 1C). We elucidated the cell type identity of clusters by examining marker genes and comparing them to those in previous studies. For example, cluster 7 (C7) and C9 were defined as hepatocyte with high expression of *leg1.1* and *bhmt*. C50 partly derived from zebrafish embryos and was identified as primitive hepatocyte with additional high level of *dao.1* and *acot17*. Compared with C7, C9, and C50 showed a closer spatial relationship in the t-SNE map. Similar to human and mouse liver cell atlas, the heterogeneity of zebrafish hepatocyte may imply zonation features at the single-cell level (Aizarani et al., 2019). Epithelial cells (C6, C8, C14, C28, and C35) were composed of multiple tissues such as caudal fin, eye, gill, muscle and skin, suggesting the consistency of transcriptome signatures of epithelial cell in different tissues. Conversely, innate immune cells of skin (C58) and intestine (C63) were identified as two independent subgroups, indicating specific tissue residency and function execution (Figures 1B,C and Supplementary Figure 1B). We then performed sub-clustering analysis for each of the 63 major clusters and generated a hierarchy of more than 600 cell-type sub-clusters (Figure 1D). Together, we clustered the expression profiles of the individual cells and constructed a comprehensive cell type landscape for zebrafish.

### Cellular Heterogeneity in Embryo and Adult Tissues

**FIGURE 2:**
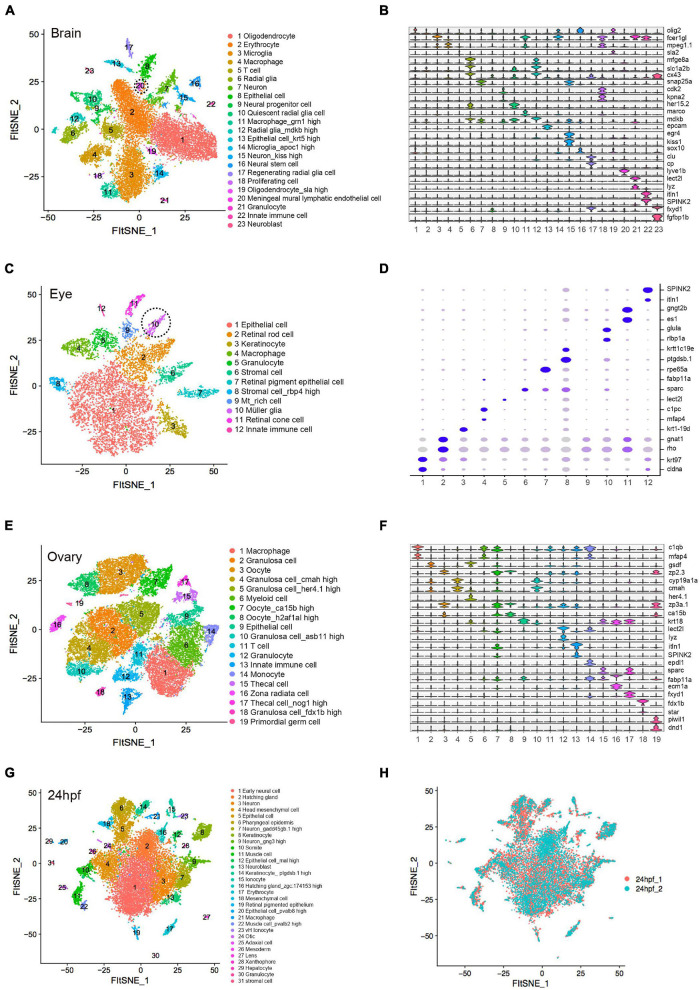
Cellular heterogeneity in embryo and adult tissues. **(A)** t-SNE map of zebrafish brain single-cell data. Cells are colored by cell-type cluster. **(B)** Violin plot showing representative gene expression in each cluster of zebrafish brain. **(C)** t-SNE map of zebrafish eye single-cell data. Cells are colored by cell-type cluster. **(D)** Dot plot showing representative gene expression in each cluster of zebrafish eye. **(E)** t-SNE map of zebrafish ovary single-cell data. Cells are colored by cell-type cluster. **(F)** Violin plot showing representative gene expression in each cluster of zebrafish ovary. **(G)** t-SNE map of zebrafish embryo (24 hpf) single-cell data. Cells are colored by cell-type cluster. **(H)** t-SNE map of zebrafish embryo (24 hpf) single-cell data. Cells are colored by different batches. hpf, hours post-fertilization.

t-SNE analysis and differential gene expression analysis for each specific organ type were performed (Figure 2, Supplementary Figure 2, and Supplementary Table 2). The brain is the most complex organ that serves as the center of the nervous system in all vertebrates, controlling over the other organs of the body (Raj et al., 2018). Similar to the previous single-cell analysis of adult zebrafish brain (Alemany et al., 2018; Spanjaard et al., 2018), our data identified four major cell groups. Two neuron sub-clusters (C7 and C15) expressed high level of *snap25a, syt1a* and *kiss1*. C6 and C12 were defined as radial glia expressing *cx43* and *fabp7a*. Gene expression of C1 and C19 encoded the classic myelin associated protein, a major constituent of the myelin sheath of oligodendrocyte in the nervous system. C3 and C14 were identified as microglia with high expression of *apoeb* (Figures 2A,B). Notably, our data additionally identified neuro precursor cells, such as C9 with high level of *neurod1*, *hes6* (Weng et al., 2019) and C10 as quiescent radial glia expressing *ascl1a*, *her15.2* and *her4.1* (Chapouton et al., 2011). Mural lymphatic endothelial cells (C20) were defined with high level of *lyve1b* (Supplementary Figure 3A). Functional experiments verified its biological significance of regulating meningeal angiogenesis in zebrafish brain (Bower et al., 2017).

By analyzing adult zebrafish eye, we defined 12 distinct clusters with specific molecular markers (Figure 2C). Similar to previous single-cell analysis of adult eye (Alemany et al., 2018), retinal rod cells (C2) and retinal cone cells (C11) were identified. Additionally, we defined C10 as müller glia, a type of retinal glial cell with high expression of *ascl1a*, *glula* and *rlbp1a* (Supplementary Figure 3C), which would be induced to dedifferentiate and produce multipotent neuronal progenitor cells in damage retina models (Raymond et al., 2006). Epithelial cells (C1 and C7), stromal cells (C6 and C8) and immune cells (C5 and C12) were also detected in zebrafish eye single-cell data (Figure 2D).

Similarly, we identified 19 different cell types in ovary. C3, C7, and C8 were defined as oocyte due to its specific expression of *zp2.3* and *zp3a.1*. C2, C4, C5, C10, and C18 represented five types of granulosa cell with high expression of *gsdf, cmah, her4.1, cyp19a1a*, and *fdx1b*, respectively (Supplementary Figure 3B). C19 was identified as primordial germ cell with high level of *dnd1* and *piwil1* (Leu and Draper, 2010; Dai et al., 2015) (Figures 2E,F). Moreover, 31 cell subpopulations were identified in zebrafish pharyngula stage (24 hpf), when it possesses the classic vertebrate bauplan. C1, C3, C7, C9, and C13 represented neuro-related cell types that highly expressed *neurog1* and *snap25a*. Epithelial-associated cells corresponded to C5, C6, C8, C12, C14, and C20 with high level of *epcam* and *krt18*. C11 and C22 were muscle cells expressing *myl1* and *mylpfa*. C26 was identified as mesoderm cells that expressed *etv2* and *lmo2* (Farnsworth et al., 2019). In addition, we detected ionocytes (C15 and C23), immune cells (C17, C21, and C30), otic (C24), lens (C27), xanthophore (C28) and hepatocytes (C29) (Figure 2G). Transcriptomic profiling showed no significant differences among diverse batches of zebrafish embryonic single-cell dataset, indicating that the cell identity reflected biological differences between cells rather than technical variations (Figure 2H). The single-cell resources of zebrafish embryo and adult tissues are publicly available at http://bis.zju.edu.cn/ZCA/.

### Cross-Species Analysis of Cell-Type Similarity

**FIGURE 3:**
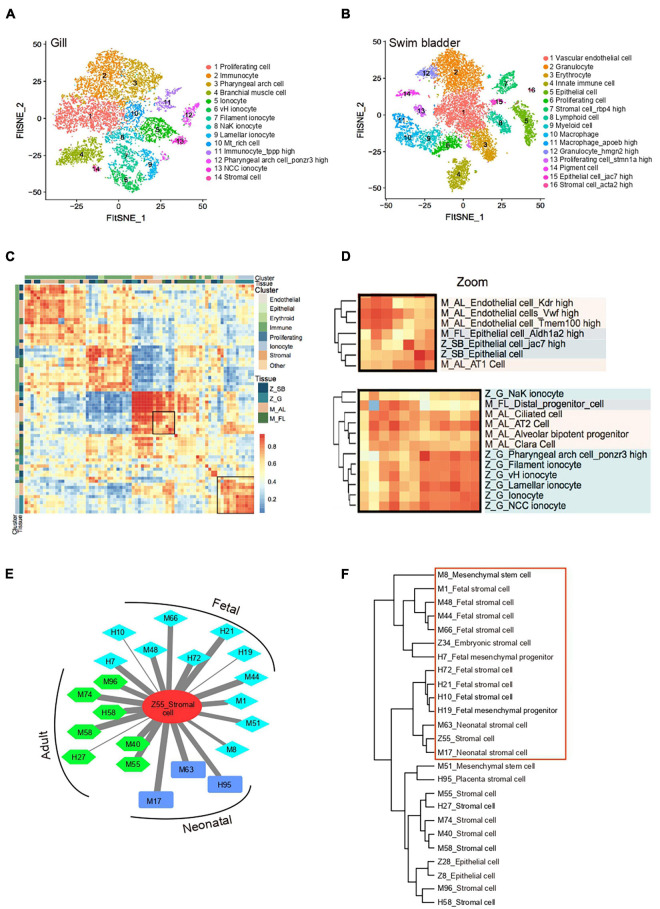
Cross-species analysis of cell-type similarity. **(A)** t-SNE map of zebrafish gill single-cell data. Cells are colored by cell-type cluster. **(B)** t-SNE map of zebrafish swim bladder single-cell data. Cells are colored by cell-type cluster. **(C)** Comparative transcriptome analysis of cell similarity between zebrafish gill, swim bladder and mouse lung. Red corresponds to a high correlation; blue and white correspond to low correlation. **(D)** Comparative transcriptome analysis of cell similarity between gill ionocytes and mouse lung epithelial cells. Zoom-in view of the region highlighted by a solid line in **(C)**. Red corresponds to a high correlation; blue and white correspond to low correlation. Z_SB, zebrafish swim bladder; Z_G, zebrafish gill; M_AL, mouse adult lung; M_FL, mouse fetal lung. **(E)** A cell–cell correlation network between zebrafish stromal cell and human, mouse fetal, neonatal, adult stromal cells. Thick lines indicate high correlation; thin lines indicate low correlation. H, human; M, mouse; Z, zebrafish. **(F)** Hierarchical clustering identified correlation of human, mouse and zebrafish stromal cells.

Single-cell transcriptomics offers an opportunity for comprehensive cross-species and cross-tissues analysis of cell types. The lung is the primary organ for gas exchange in mammals. The swim bladder and gill are specialized organs in teleosts that regulate respiration. Whether the lung evolves from the gill or the swim bladder is still a controversy based on morphological evidences (Perry and Sander, 2004; Zheng et al., 2011). A recent study reported that the cell types could be proposed as “evolutionary units” in comparative cell biology (Wang et al., 2021). To infer the evolutionary relationship of respiratory system at the single-cell level, we extracted orthologous genes and performed cross-species clustering analysis of zebrafish gill (Z_G), swim bladder (Z_SB) and mouse adult lung (M_AL), fetal lung (M_FL) (Han et al., 2018) (Figures 3A,B and Supplementary Table 3). As revealed by the heatmap and circos plot, the gene expression patterns of major cell types showed strong correlations between mouse and zebrafish, such as immune cells, stromal cells and proliferating cells (Figure 3C and Supplementary Figure 4A). Notably, based on transcriptome distances that were evaluated by correlation coefficient, we found that adult mouse lung alveolar type 1 (AT1) cells specialized for gas exchange displayed strong correlations with zebrafish swim bladder epithelial cells, while other epithelial cells shared transcriptional similarity with gill ionocytes (Figure 3D and Supplementary Figure 4B). Recent studies identified Foxi1+ pulmonary ionocytes in mouse lung, a rare cell type in the conducting airway at single-cell resolution (Plasschaert et al., 2018). We propose that a gene’s function is strongly predictive of conservation in gene expression program; the evolution of cell-type regulatory network may be independent of tissue evolution. Taken together, our single-cell transcriptomes demonstrated the conservation and divergence of cell types between zebrafish swim bladder, gill and mouse lung, providing potential resources to infer the cell-type evolutionary relationship.

Unlike mammals, zebrafish adult tissues exert powerful regenerative ability (Gemberling et al., 2013). To understand the potential cellular mechanism, we calculated the correlation of cell-type between the zebrafish cell landscape and human cell landscape, mouse cell atlas, axolotl limbs regenerative landscape (Han et al., 2018, 2020; Leigh et al., 2018). scRNA-seq datasets revealed a well-conserved cellular architecture that enables matching of homologous cell types between zebrafish and other species, such as embryonic cell, oligodendrocyte, enterocyte, epithelial cell and granulocyte etc. (Supplementary Table 3). Notably, we found that zebrafish adult stromal cells showed a strong correlation with both human and mouse fetal stromal cells, resembling zebrafish embryonic stromal cells (Figure 3E and Supplementary Figure 4C). In cross-species clustering analysis, adult zebrafish stromal cells were clearly close to fetal and neonatal mammalian stromal cells (Figure 3F). Moreover, zebrafish adult stromal cells were strongly associated with fibroblast-like blastema in axolotl limbs regenerative landscape (Supplementary Figure 4D). Taken together, adult zebrafish stromal cells are intrinsically different from the adult human and mouse stromal cells. They possess a progenitor-like phenotype that is only seen in fetal mammalian tissues. This may help to explain stronger regenerative potentials in the adult zebrafish tissues when compared to higher organisms.

### Characteristic of Blastema Cells During Caudal Fin Regeneration

**FIGURE 4:**
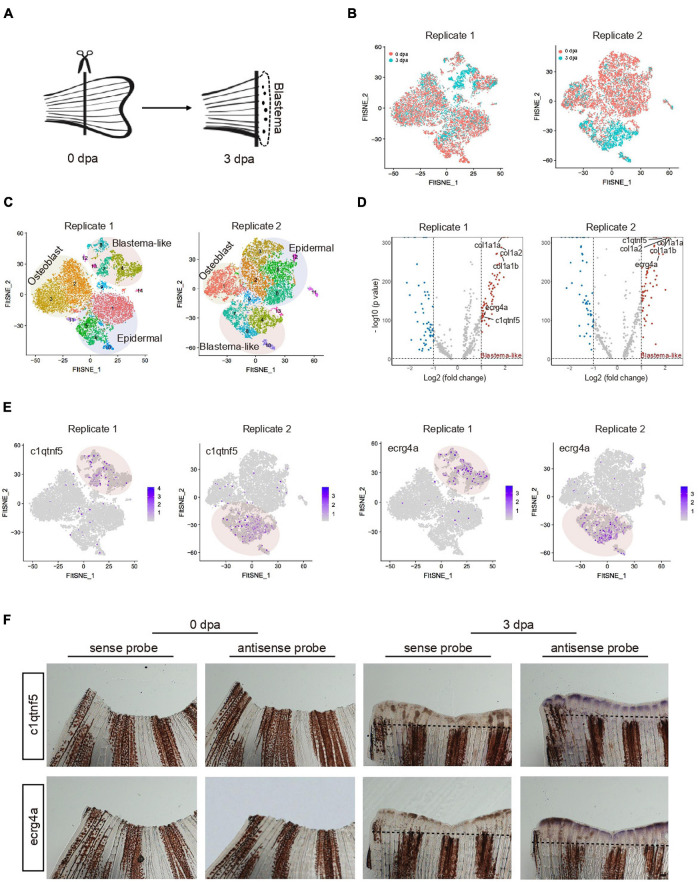
Characteristic of blastema cells during caudal fin regeneration. **(A)** A schematic of amputated primary caudal fin and blastema formed after 3 days. **(B)** t-SNE map of zebrafish caudal fin regeneration single-cell data from two independent biological replicates termed Replicate 1 (left) and Replicate 2 (right). Cells are colored by 0 and 3 dpa. **(C)** t-SNE map of zebrafish caudal fin regeneration single-cell data from Replicate 1 (left) and Replicate 2 (right). Cells are colored by cell-type cluster. **(D)** Volcano plot showing differentially expressed genes in blastema cells from Replicate 1 (left) and Replicate 2 (right). **(E)** Feature plot showing high expression of *c1qtnf5* and *ecrg4a* in caudal fin regeneration single-cell dataset from Replicate 1 and Replicate 2. **(F)** Whole mount *in situ* hybridization against marker genes with sense and antisense probe in caudal fin at 0 and 3 dpa. Dashed lines indicate the amputation planes. *n* = 3 independent experiments. dpa, days post-amputation. scale bars, 200 μm.

To investigate the regenerative capability of the zebrafish system, we then focused on zebrafish caudal fin ontogeny and its regeneration process. scRNA-seq was performed to profile uninjured fin and outgrowth of regenerated blastema cells at 3 days post-amputation (dpa) from two independent biological replicates termed replicate 1 and replicate 2 (Poss et al., 2003) (Figure 4A). An independent analysis of the two biological replicates revealed that the results of both experiments strongly overlapped. Transcriptomic profiling of uninjured (0 dpa) and regenerated fin (3 dpa) contributed to nearly all cell clusters, suggesting that the cell identity was unaffected by batch effect (Figure 4B). Three major cell types (osteoblast, epidermal and blastema-like cell) were identified based on gene expression signatures (Supplementary Table 4). Among them, blastema-like cells were mainly composed of four subpopulations. In replicate 1, C4 was identified as stromal-related cells with high expression of *col1a1a* and *col1a1b* (C7 in replicate 2), C8 expressed high level of fibroblast growth factor binding protein (*fgfbp2a*) (C10 in replicate 2), C7 and C13 differed mainly in higher expression of proliferation genes (C4 and C8 in replicate 2), such as *hmgn2*, *pcna* (Figure 4C).

To further reveal the unique characteristic of blastema-like cells, we next analyzed the differentially expressed genes (DEGs) between uninjured (0 dpa) and regenerated fin (3 dpa). Upregulated markers in blastema-like cells during caudal fin regeneration were identified (Figure 4D). Extracellular matrix genes, such as *col1a1a* and *col1a2*, showed significantly enhanced expression, indicating that stromal cells mainly participated in blastema formation. The result is in accordance with previous findings that connective tissues transit to a blastema state during adult axolotl and frog limb regeneration (Gerber et al., 2018; Lin et al., 2021). Besides, blastema were enriched in cells expressing *c1qtnf5*, *ecrg4a, and1, clu*, and *fgfbp2a*, with confirmation of their localization by *in situ* hybridization (Figures 4E,F and Supplementary Figures 5A,B). We found that *c1qtnf5* and *ecrg4a* were only expressed in outgrowth blastema involved in regulation of cell proliferation, which is consistent with zebrafish mantle cells (hair-cell progenitors) that express high levels of *c1qtnf5* and *ecrg4a* during hair cell regeneration (Steiner et al., 2014). *clu*, a marker of mouse intestine revival stem cell (Ayyaz et al., 2019), exhibited potential regulation function during zebrafish tissue repair. Together, our data illuminated molecular characteristics of blastema cells during zebrafish caudal fin regeneration at single-cell resolution.

### Genetic Regulation During Tissue Regeneration

**FIGURE 5:**
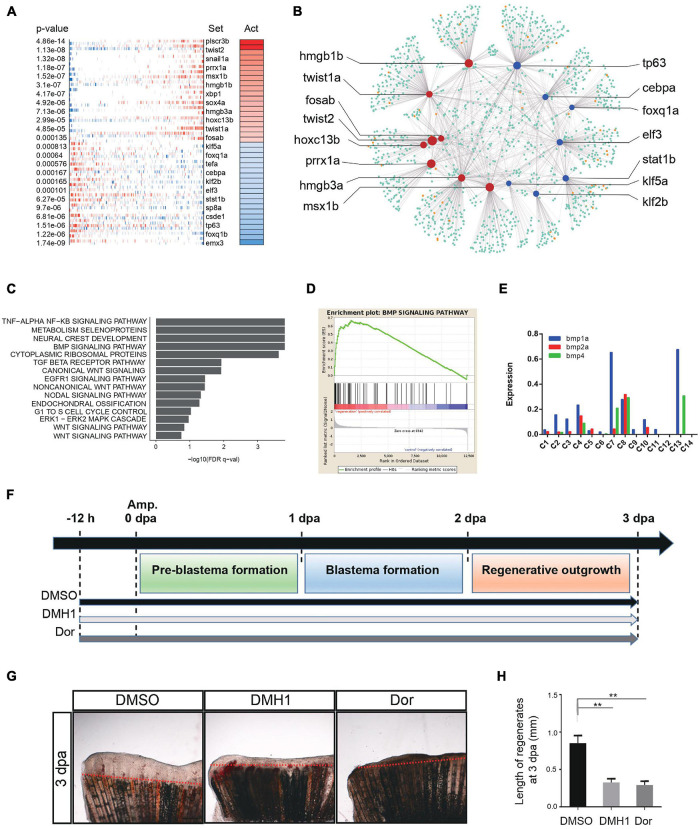
Genetic regulation during tissue regeneration. **(A)** Virtual inference of protein-activity by enriched regulon analysis in caudal fin Replicate 1. Red represents activated transcription factors; blue indicates repressed transcription factors. Act, activation. **(B)** A gene-gene correlation network of regeneration module. Red corresponds to high activation transcription factors; blue corresponds to low activation transcription factors; orange corresponds to co-factor genes; green corresponds to targeted genes. **(C)** Gene set enrichment analysis of caudal fin regeneration module in Replicate 1. **(D)** Gene set enrichment analysis between caudal fin regeneration module and non-regeneration module in Replicate 1. **(E)** Histogram showing BMP signaling pathway-related gene expression of each subgroup in caudal fin regeneration from Replicate 1. **(F)** Scheme of DMH1 and dorsomorphin treatment from –12 h to 3 dpa. dpa, days post-amputation. **(G,H)** DMH1 and dorsomorphin (Dor) treatment both significantly inhibited fin regeneration from –12 h to 3 dpa (pre-blastema formation, blastema formation, and regenerative outgrowth stages), when compared to DMSO treatment. Red dashed lines indicate the amputation planes. **p < 0.01 by Student’s *t*-test. Error bars represent the standard error of 4 independent experiments. dpa, days post-amputation; scale bars, 500 μm in **(G)**.

Transcription factors (TFs) directly interpret the genome, exerting control over processes that specify cell types and controlling specific pathways (Lambert et al., 2018). To understand the genetic regulation during zebrafish caudal fin regeneration, we next focused on the activity of TFs by enriched regulon analysis. We performed weighted correlation network analysis (WGCNA) to look for clusters (modules) of highly correlated genes (Langfelder and Horvath, 2008). We constructed a gene co-expression network in zebrafish caudal fin regeneration single-cell dataset. Blastema-like cells (C4, C7, C8, and C13 in replicate 1) were integrated into one regeneration gene module, and other clusters were defined as non-regenerative module (Supplementary Figure 6A). Virtual inference of protein-activity by enriched regulon analysis (VIPER) was then performed to infer the relative activity of a regulatory TF based on the enrichment of its most closely-regulated targets on a given gene expression signature (Alvarez et al., 2016). Comparing the regeneration and non-regenerative gene modules, we identified high activity TFs regulating regeneration progress, such as *twist1a*, *prrx1a*, *msx1b, fosab*, etc., as well as low activity TFs such as *tp63*, *elf3* and *klf2b* (Figures 5A,B, Supplementary Figure 6B, and Supplementary Table 5). Previous studies found that *twist1a* and *prrx1a* play a vital role in axolotl limb regeneration, *msx1b* and *fosab* are essential for cell proliferation and differentiation (Gerber et al., 2018; Hirsch et al., 2018).

Next, we sought to look for key signaling pathways that regulate tissue regeneration. In the gene enrichment analysis, bone morphogenetic protein (BMP) signaling pathway was found to be strongly associated with the zebrafish caudal fin regenerative process (Figure 5C and Supplementary Figure 6C), exhibiting significant difference between regenerative and non-regenerative module (Figure 5D and Supplementary Figure 6D). The BMP signaling pathway was mainly regulated by blastema cells, in which *bmp4* was only expressed in 4 clusters of blastema, *bmp2a* was enriched in C4 and C8, and *bmp1a* was highly expressed in C7 and C13 (Figure 5E). Besides, ligand-receptor map was constructed to reveal cell–cell interactions during caudal fin regeneration (Supplementary Figure 6E). We found that the 4 clusters of blastema cell were at the center of the network, regulating the regeneration process. Previous studies have reported that BMP signaling pathway contributed to zebrafish cardiomyocyte regeneration (Wu et al., 2016) and whole body regeneration in acoels (Srivastava et al., 2014). To further examine the function of BMP signaling during caudal fin repair, adult zebrafish were treated with two specific BMP inhibitors, DMH1 and dorsomorphin (Dor), 12 h before amputation (−12 h) to 3 dpa (Figure 5F). The results showed that DMH1 and Dor significantly inhibited caudal fin regeneration compared to DMSO, suggesting that the BMP signaling pathway plays crucial roles during zebrafish caudal fin repair (Smith et al., 2006) (Figures 5G,H). Summarily, insights into genetic regulation and signaling pathways involved in caudal fin regeneration have clear implications for future prospects in tissue regenerative engineering.

## Discussion

Here, we used Microwell-seq to generate a zebrafish cell landscape. At the current stage, although the sequencing relatively shallow, the zebrafish cell landscape data certainly allows for the separation of major cell types in the zebrafish system. After the cell clustering, reads from the same cell type can then be aggregated for deeper investigation of genetic regulation. Single-cell transcriptome analysis has already been applied in zebrafish embryo, larvae, and in brain, kidney, eye, caudal fin, heart, liver and pancreas of adult fish (Tang et al., 2017; Spanjaard et al., 2018; Farnsworth et al., 2019). When compared to available single-cell data of zebrafish tissues, we have covered a more comprehensive tissue types in terms of the breath of the analysis. In pharyngula stage (24 hpf) single-cell analysis, neural cell, hatch gland, epithelial cell, muscle, ionocyte, macrophage, lens and otic were identified in both our study and a previously published work (Wagner et al., 2018). Moreover, our single-cell data additionally identified xanthophore and hepatocyte which is missing in Wagner’s research. In zebrafish kidney, Tang et al. (2017) identified 14 clusters of kidney and hematopoietic cells, while we identified 18 clusters in our microwell-seq dataset of zebrafish kidney. Among these clusters, nephron epithelial cell, proximal tubular cell, distal tubular cell, vascular endothelial cell, mucin cell, HSC, macrophage, erythrocyte, and blood progenitors are highly comparable. The proliferating cell and innate immune cell identified in our study correspond to the kidney progenitor cell and lymphoid cell in Tang’s research, respectively. Interestingly, we found a fraction of immune-active epithelial cells expressing high level of *mal* in zebrafish caudal fin, skin, and swim bladder, indicating an important role of epithelial cells involved in immune responses (Supplementary Figure 3D) (Schleimer et al., 2007; Han et al., 2020). Our work is by no mean a complete representation of all zebrafish cell types, but we constructed an initial draft to create an organism-wide cellular hierarchy for the adult zebrafish.

Single-cell transcriptomics offers an opportunity for comprehensive cross-species and cross-tissues analysis of cell types. A recent study reported that the cell-type could be proposed as “evolutionary units” in comparative cell biology (Wang et al., 2021). In the current study, we propose that the different epithelial cells in mammal lung may evolve from different organs in zebrafish including swim bladder and gill. In other words, the evolution of cell-type regulatory network may be independent and superior to tissue evolution.

Unlike mammals, zebrafish is a model system with an amazing capacity for regeneration (Marques et al., 2019). To understand the potential cellular mechanism, we performed cross-species analysis of cell-type similarity between the zebrafish cell landscape and human cell landscape, mouse cell atlas. The results showed that adult zebrafish stromal cells shared strong similarity to human and mouse fetal stromal cells. This may help to explain stronger regenerative potentials in the adult zebrafish tissues when compared to higher organisms.

During caudal fin regeneration, cells near the amputation plane accumulate into a distinctive tissue called the blastema. Blastema formation is a comprehensive process, comprising various different cell dedifferentiation, proliferation and redifferentiation to rebuild the missing fin structures (Pfefferli and Jaźwińska, 2015). In this study, our single-cell analysis of blastema population showed unique transcriptional signatures and key signaling pathways involved in caudal fin repair. The two biological replicates show high similarities in both blastema gene expression and TF regulation. Previous study revealed that zebrafish mantle cells expressed high level of *c1qtnf5* and *ecrg4a* during hair cell regeneration (Steiner et al., 2014). Similarly, we found *c1qtnf5* and *ecrg4a* were involved in the formation of blastema during caudal fin regeneration, and verified it by *in situ* hybridization (Figure 4F). Focused on the genetic regulation, we found previously identified key blastema marker genes, including the muscle segment homeobox family member *msx1b* and bone development and regeneration TF *twist1a* (Hou et al., 2020). During axolotl limb regeneration, there is a population of relatively homogenous progenitor cells similar to the embryonic state (Gerber et al., 2018). Hmgb is a well-conserved nuclear protein and plays vital role in the development of zebrafish pectoral fin buds and mouse forelimb buds, which also regulates caudal fin regeneration found in our study (Itou et al., 2011). However, *cx43*, a gap junction protein required to build the right fin length, was not identified in this study (Figure 5B). We believe that combination of other molecular tools, such as lineage tracing and transplantation-based functional assays, will further broaden our knowledge on the origin of blastema cells, the initial signals of cell regeneration, and the impact of microenvironment in the near future (Lin et al., 2021). A deep knowledge on the single-cell data of zebrafish tissue repair would inspire new strategies for controlling tissue regeneration in mammals.

## Conclusion

We present a zebrafish cell landscape with single-cell composition for many tissues that have not been well characterized. We reveal a unique molecular and cellular phenotype during caudal fin regeneration. Our single-cell datasets improve our understanding of the zebrafish and provide a valuable reference for future studies.

## Data Availability Statement

The datasets presented in this study can be found in online repositories. The names of the repository/repositories and accession number(s) can be found in the article/Supplementary Material.

## Ethics Statement

The animal study was reviewed and approved by the Ethics Committee of the Zhejiang University Laboratory Animal Center.

## Author Contributions

GG, XH, and JP were responsible for the conception, design, and study supervision. MJ, HC, CG, and YL were responsible for the development of methodology, analysis, and experiments. YX, WE, LM, JW, and QG performed the bioinformatics analysis and constructed the website of zebrafish cell landscape. All authors read and approved the final manuscript.

## Conflict of Interest

The authors declare that the research was conducted in the absence of any commercial or financial relationships that could be construed as a potential conflict of interest.

## Publisher’s Note

All claims expressed in this article are solely those of the authors and do not necessarily represent those of their affiliated organizations, or those of the publisher, the editors and the reviewers. Any product that may be evaluated in this article, or claim that may be made by its manufacturer, is not guaranteed or endorsed by the publisher.

## Footnotes

1
http://www.kbspheretech.com/

2
http://mccarrolllab.org/dropseq/

3
https://toppgene.cchmc.org

4
https://github.com/califano-lab/ARACNe-AP

5
http://bioconductor.org/packages/release/bioc/html/viper.html

6
http://software.broadinstitute.org/gsea

## Supplementary Material

Supplementary Figure 1 Constructing a zebrafish cell landscape using microwell-seq. **(A)** Number of cells currently processed in zebrafish cell landscape. **(B)** The cluster contribution bar charts in zebrafish cell landscape.

Supplementary Figure 2 Cellular heterogeneity in embryo and adult tissues. t-SNE map of zebrafish embryo (72 hpf), blood, caudal fin, heart, intestine, pancreas, kidney, liver, muscle, skin, spleen, testis single-cell data. Cells are colored by cell-type cluster. hpf, hours post-fertilization.

Supplementary Figure 3 Signatures of gene expression in adult tissues. **(A)** Feature plot showing representative gene expression in zebrafish brain. **(B)** Feature plot showing representative gene expression in zebrafish ovary. **(C)** Feature plot showing representative gene expression in zebrafish eye. **(D)** Dot plot showing the expression of *mal* in zebrafish caudal fin, skin, and swim bladder.

Supplementary Figure 4 Cross-species analysis of cell-type similarity. **(A)** Circos plot showing the similarity of cell types in zebrafish gill, swim bladder and mouse lung. Paired cell types with average AUROC scores greater than 0.8 are connected by lines. **(B)** A cell-type correlation network between lung AT1 cell and swim bladder epithelial cell, gill ionocyte and other lung epithelial cell. Thick lines indicate high correlation; thin lines indicate low correlation. Z_SB, zebrafish swim bladder; Z_G, zebrafish gill; M_AL, mouse adult lung; M_FL, mouse fetal lung. **(C)** A cell–cell correlation network between zebrafish embryonic stromal cells and human, mouse fetal, neonatal, adult stromal cells. Thick lines indicate high correlation; thin lines indicate low correlation. H, human; M, mouse; Z, zebrafish. **(D)** Correlation between zebrafish embryonic stromal cell, stromal cell, epithelial cell and axolotl limbs regenerative landscape. Red indicates high correlation; blue and yellow indicate low correlation. Z, zebrafish.

Supplementary Figure 5 Characteristic of blastema cells during caudal fin regeneration. **(A)** Feature plot showing high expression of *and1, clu*, and *fgfbp2a* in caudal fin regeneration single-cell dataset from Replicate 1 and Replicate 2. **(B)** Whole mount *in situ* hybridization against marker genes with sense and antisense probe in caudal fin at 0 and 3 dpa. Dashed lines indicate the amputation planes. *n* = 3 independent experiments. dpa, days post-amputation. scale bars, 200 μm.

Supplementary Figure 6 Genetic regulation during tissue regeneration. **(A)** Heatmap showing correlation between caudal fin regeneration modules and cell types from Replicate 1. Red corresponds to a high correlation; blue and white correspond to low correlation. **(B)** Virtual inference of protein-activity by enriched regulon analysis in caudal fin Replicate 2. Red represents activated transcription factors; blue indicates repressed transcription factors. Act, activation. **(C)** Gene set enrichment analysis of caudal fin regeneration module in Replicate 2. **(D)** Gene set enrichment analysis between caudal fin regeneration module and non-regeneration module in Replicate 2. **(E)** Ligand and receptor analysis of caudal fin regeneration by CellPhoneDB. The colors represent cell types; line thickness indicates the degree of association between cell types.

Supplementary Table 1 Constructing a zebrafish cell landscape using microwell-seq. Sheet 1: Basic information about tissues processed in the zebrafish cell landscape. The table contains the dissociation, concentration, digestion time of the tissues. Sheet 2: Differentially expressed genes detected in 63 zebrafish cell types from zebrafish cell landscape. *P*-values were calculated by the Wilcoxon-Rank-Sum test. (n: gene name, l: logFc, s:score, p: *P*-value). Sheet 3: The number of sub-cluster in 63 zebrafish cell types. Sheet 4: The oligonucleotide sequences used in single cell mRNA-seq library construction.

Supplementary Table 2 Cellular heterogeneity in embryo and adult tissues. Differentially expressed genes detected in each cell type for all tissues in zebrafish cell landscape datasets. Yellow labels indicate specific marker genes of cell clusters. Genes are selected by log foldchange > 0.25, Bonferroni-adjusted *p*-value < 0.1, expressed in at least 15% of cells in either population (Seurat FindAllMarkers). Log fold change is calculated as arithmetic mean of log10 cpm values of one population minus the arithmetic mean of log10 cpm values of the second, and fold change is 10log_foldchange. *P*-values were calculated by the Wilcoxon rank sum test. The last sheet showing cell barcodes of each cell in zebrafish tissues.

Supplementary Table 3 Cross-species analysis of cell-type similarity. Sheet 1: Cell type annotation of zebrafish gill, swim bladder and mouse lung. Sheet 2: The correlation coefficient of cell types among zebrafish gill, swim bladder and mouse lung. Sheet 3: The correlation of cell-type among zebrafish cell landscape and human, mouse cell landscape. Sheet 4: The correlation of cell-type among zebrafish cell landscape and axolotl limbs regenerative landscape.

Supplementary Table 4 Characteristic of blastema cells during caudal fin regeneration. Differentially expressed genes detected in each cell type for caudal fin regeneration. Genes are selected by log foldchange > 0.25, Bonferroni-adjusted *p*-value < 0.1, expressed in at least 15% of cells in either population (Seurat FindAllMarkers). Log fold change is calculated as arithmetic mean of log10 cpm values of one population minus the arithmetic mean of log10 cpm values of the second, and fold change is 10log_foldchange. P-values were calculated by the Wilcoxon rank sum test.

Supplementary Table 5 Genetic regulation during tissue regeneration. Sheets 1 and 2: The transcription factor-activity of caudal fin regeneration module. Sheet 3: Activated and repressed transcription factors in caudal fin regeneration module, as well as its target genes. Sheets 4 and 5: Gene set enrichment analysis of caudal fin regeneration module.

## Abbreviations

scRNA-seq: single-cell RNA sequencing
UMI: unique molecular identifier
t-SNE: t-Distributed stochastic neighbor embedding
hpf: hours post-fertilization
dpa: days post-amputation
BMP: bone morphogenetic protein
AUROC: area under the receiver operator characteristic curve
DGE: digital gene expression
PCA: principal component analysis
TF: transcription factor
GEO: gene expression omnibus
WGCNA: weighted correlation network analysis
VIPER: virtual inference of protein-activity by enriched regulon analysis
NES: normalized enrichment score
GSEA: gene set enrichment analysis
GO: gene ontology
DEG: differentially expressed gene
WISH: whole mount *in situ* hybridization.

## References

[B1] Aizarani N. Saviano A. Sagar G. Mailly L. Durand S. Herman J. S. (2019). A human liver cell atlas reveals heterogeneity and epithelial progenitors. *Nature* 572 199–204. 10.1038/s41586-019-1373-2 31292543 PMC6687507

[B2] Alemany A. Florescu M. Baron C. S. Peterson-Maduro J. van Oudenaarden A. (2018). Whole-organism clone tracing using single-cell sequencing. *Nature* 556:108. 10.1038/nature25969 29590089

[B3] Alvarez M. J. Shen Y. Giorgi F. M. Lachmann A. Ding B. B. Ye B. H. (2016). Functional characterization of somatic mutations in cancer using network-based inference of protein activity. *Nat. Genet.* 48 838–847. 10.1038/ng.3593 27322546 PMC5040167

[B4] Ayyaz A. Kumar S. Sangiorgi B. Ghoshal B. Gosio J. Ouladan S. (2019). Single-cell transcriptomes of the regenerating intestine reveal a revival stem cell. *Nature* 569 121–125. 10.1038/s41586-019-1154-y 31019301

[B5] Aztekin C. Hiscock T. W. Marioni J. C. Gurdon J. B. Simons B. D. Jullien J. (2019). Identification of a regeneration-organizing cell in the Xenopus tail. *Science* 364 653–658. 10.1126/science.aav9996 31097661 PMC6986927

[B6] Bower N. I. Koltowska K. Pichol-Thievend C. Virshup I. Paterson S. Lagendijk A. K. (2017). Mural lymphatic endothelial cells regulate meningeal angiogenesis in the zebrafish. *Nat. Neurosci.* 20:774. 10.1038/nn.4558 28459441

[B7] Buettner F. Natarajan K. N. Casale F. P. Proserpio V. Scialdone A. Theis F. J. (2015). Computational analysis of cell-to-cell heterogeneity in single-cell RNA-sequencing data reveals hidden subpopulations of cells. *Nat. Biotechnol.* 33:155. 10.1038/nbt.3102 25599176

[B8] Celniker S. E. Dillon L. A. Gerstein M. B. Gunsalus K. C. Henikoff S. Karpen G. H. (2009). Unlocking the secrets of the genome. *Nature* 459:927. 10.1038/459927a 19536255 PMC2843545

[B9] Chapouton P. Webb K. J. Stigloher C. Alunni A. Adolf B. Hesl B. (2011). Expression of hairy/enhancer of split genes in neural progenitors and neurogenesis domains of the adult zebrafish brain. *J. Comp. Neurol.* 519 1748–1769. 10.1002/cne.22599 21452233

[B10] Crow M. Paul A. Ballouz S. Huang Z. J. Gillis J. (2018). Characterizing the replicability of cell types defined by single cell RNA-sequencing data using MetaNeighbor. *Nat. Commun.* 9:884. 10.1038/s41467-018-03282-0 29491377 PMC5830442

[B11] Dai X. Jin X. Chen X. He J. Yin Z. (2015). Sufficient numbers of early germ cells are essential for female sex development in zebrafish. *PLoS One* 10:e0117824. 10.1371/journal.pone.0117824 25679390 PMC4332673

[B12] Dobin A. Davis C. A. Zaleski C. Schlesinger F. Drenkow J. Chaisson M. (2012). STAR: ultrafast universal RNA-seq aligner. *Bioinformatics* 29 15–21. 10.1093/bioinformatics/bts635 23104886 PMC3530905

[B13] Farnsworth D. R. Saunders L. M. Miller A. C. (2019). A single-cell transcriptome atlas for zebrafish development. *Dev. Biol.* 459 100–108. 10.1016/j.ydbio.2019.11.008 31782996 PMC7080588

[B14] Farrell J. A. Wang Y. Riesenfeld S. J. Shekhar K. Regev A. Schier A. F. (2018). Single-cell reconstruction of developmental trajectories during zebrafish embryogenesis. *Science* 360:eaar3131. 10.1126/science.aar3131 29700225 PMC6247916

[B15] Fincher C. T. Wurtzel O. de Hoog T. Kravarik K. M. Reddien P. W. (2018). Cell type transcriptome atlas for the planarian Schmidtea mediterranea. *Science* 360:eaaq1736. 10.1126/science.aaq1736 29674431 PMC6563842

[B16] Gemberling M. Bailey T. J. Hyde D. R. Poss K. D. (2013). The zebrafish as a model for complex tissue regeneration. *Trends Genet.* 29 611–620. 10.1016/j.tig.2013.07.003 23927865 PMC3812420

[B17] Gerber T. Murawala P. Knapp D. Masselink W. Schuez M. Hermann S. (2018). Single-cell analysis uncovers convergence of cell identities during axolotl limb regeneration. *Science* 362:eaaq0681. 10.1126/science.aaq0681 30262634 PMC6669047

[B18] Gu Z. Gu L. Eils R. Schlesner M. Brors B. (2014). circlize implements and enhances circular visualization in R. *Bioinformatics* 30 2811–2812. 10.1093/bioinformatics/btu393 24930139

[B19] Gupta I. Collier P. G. Haase B. Mahfouz A. Joglekar A. Floyd T. (2018). Single-cell isoform RNA sequencing characterizes isoforms in thousands of cerebellar cells. *Nat. Biotechnol.* 36 1197–1202. 10.1038/nbt.4259 30320766

[B20] Han X. Wang R. Zhou Y. Fei L. Sun H. Lai S. (2018). Mapping the mouse cell atlas by microwell-seq. *Cell* 173:1307. 10.1016/j.cell.2018.05.012 29775597

[B21] Han X. Zhou Z. Fei L. Sun H. Wang R. Chen Y. (2020). Construction of a human cell landscape at single-cell level. *Nature* 581 303–309. 10.1038/s41586-020-2157-4 32214235

[B22] Hirsch N. Eshel R. Bar Yaacov R. Shahar T. Shmulevich F. Dahan I. (2018). Unraveling the transcriptional regulation of TWIST1 in limb development. *Plos Genet.* 14:e1007738. 10.1371/journal.pgen.1007738 30372441 PMC6233932

[B23] Hou Y. Lee H. J. Chen Y. Ge J. Osman F. O. I. McAdow A. R. (2020). Cellular diversity of the regenerating caudal fin. *Sci. Adv.* 6:eaba2084. 10.1126/sciadv.aba2084 32851162 PMC7423392

[B24] Itou J. Taniguchi N. Oishi I. Kawakami H. Lotz M. Kawakami Y. (2011). HMGB factors are required for posterior digit development through integrating signaling pathway activities. *Dev. Dyn.* 240 1151–1162. 10.1002/dvdy.22598 21384471 PMC3081368

[B25] Klein A. M. Mazutis L. Akartuna I. Tallapragada N. Veres A. Li V. (2015). Droplet barcoding for single-cell transcriptomics applied to embryonic stem cells. *Cell* 161 1187–1201. 10.1016/j.cell.2015.04.044 26000487 PMC4441768

[B26] Lambert S. A. Jolma A. Campitelli L. F. Das P. K. Yin Y. Albu M. (2018). The human transcription factors. *Cell* 172 650–665. 10.1016/j.cell.2018.01.029 29425488 PMC12908702

[B27] Langfelder P. Horvath S. (2008). WGCNA: an R package for weighted correlation network analysis. *BMC Bioinformatics* 9:559. 10.1186/1471-2105-9-559 19114008 PMC2631488

[B28] Leigh N. D. Dunlap G. S. Johnson K. Mariano R. Oshiro R. Wong A. Y. (2018). Transcriptomic landscape of the blastema niche in regenerating adult axolotl limbs at single-cell resolution. *Nat. Commun.* 9:5153. 10.1038/s41467-018-07604-0 30514844 PMC6279788

[B29] Leu D. H. Draper B. W. (2010). The ziwi promoter drives germline-specific gene expression in zebrafish. *Dev. Dyn.* 239 2714–2721. 10.1002/dvdy.22404 20737503

[B30] Lin T. Y. Gerber T. Taniguchi-Sugiura Y. Murawala P. Hermann S. Grosser L. (2021). Fibroblast dedifferentiation as a determinant of successful regeneration. *Dev. Cell* 56 1541–1551. 10.1016/j.devcel.2021.04.016 34004152 PMC8140481

[B31] Macosko E. Z. Basu A. Satija R. Nemesh J. Shekhar K. Goldman M. (2015). Highly parallel genome-wide expression profiling of individual cells using nanoliter droplets. *Cell* 161 1202–1214. 10.1016/j.cell.2015.05.002 26000488 PMC4481139

[B32] Manoli M. Driever W. (2012). Fluorescence-activated cell sorting (FACS) of fluorescently tagged cells from zebrafish larvae for RNA isolation. *Cold Spring Harb. Protoc.* 2012:879. 10.1101/pdb.prot069633 22854565

[B33] Margolin A. A. Nemenman I. Basso K. Wiggins C. Stolovitzky G. Dalla Favera R. (2006). ARACNE: an algorithm for the reconstruction of gene regulatory networks in a mammalian cellular context. *BMC Bioinformatics* 7:S7. 10.1186/1471-2105-7-S1-S7 16723010 PMC1810318

[B34] Marques I. J. Lupi E. Mercader N. (2019). Model systems for regeneration: zebrafish. *Development* 146:dev167692. 10.1242/dev.167692 31540899

[B35] Packer J. S. Zhu Q. Huynh C. Sivaramakrishnan P. Preston E. Dueck H. (2019). A lineage-resolved molecular atlas of C. elegans embryogenesis at single-cell resolution. *Science* 365:eaax1971. 10.1126/science.aax1971 31488706 PMC7428862

[B36] Perry S. F. Sander M. (2004). Reconstructing the evolution of the respiratory apparatus in tetrapods. *Respir. Physiol. Neurobiol.* 144 125–139. 10.1016/j.resp.2004.06.018 15556097

[B37] Pfefferli C. Jaźwińska A. (2015). The art of fin regeneration in zebrafish. *Regeneration* 2 72–83. 10.1002/reg2.33 27499869 PMC4895310

[B38] Picelli S. Faridani O. R. Bjorklund A. K. Winberg G. Sagasser S. Sandberg R. (2014). Full-length RNA-seq from single cells using smart-seq2. *Nat. Protoc.* 9 171–181. 10.1038/nprot.2014.006 24385147

[B39] Plass M. Solana J. Wolf F. A. Ayoub S. Misios A. Glazar P. (2018). Cell type atlas and lineage tree of a whole complex animal by single-cell transcriptomics. *Science* 360:eaaq1723. 10.1126/science.aaq1723 29674432

[B40] Plasschaert L. W. Žilionis R. Choo-Wing R. Savova V. Knehr J. Roma G. (2018). A single-cell atlas of the airway epithelium reveals the CFTR-rich pulmonary ionocyte. *Nature* 560 377–381. 10.1038/s41586-018-0394-6 30069046 PMC6108322

[B41] Poss K. D. Keating M. T. Nechiporuk A. (2003). Tales of regeneration in zebrafish. *Dev. Dyn.* 226 202–210. 10.1002/dvdy.10220 12557199

[B42] Raj B. Wagner D. E. McKenna A. Pandey S. Klein A. M. Shendure J. (2018). Simultaneous single-cell profiling of lineages and cell types in the vertebrate brain. *Nat. Biotechnol.* 36 442–450. 10.1038/nbt.4103 29608178 PMC5938111

[B43] Raymond P. A. Barthel L. K. Bernardos R. L. Perkowski J. J. (2006). Molecular characterization of retinal stem cells and their niches in adult zebrafish. *BMC Dev. Biol.* 6:36. 10.1186/1471-213X-6-36 16872490 PMC1564002

[B44] Satija R. Farrell J. A. Gennert D. Schier A. F. Regev A. (2015). Spatial reconstruction of single-cell gene expression data. *Nat. Biotechnol.* 33 495–U206. 10.1038/nbt.3192 25867923 PMC4430369

[B45] Schleimer R. P. Kato A. Kern R. Kuperman D. Avila P. C. (2007). Epithelium: at the interface of innate and adaptive immune responses. *J. Allergy Clin. Immunol.* 120 1279–1284. 10.1016/j.jaci.2007.08.046 17949801 PMC2810155

[B46] Sebe-Pedros A. Saudemont B. Chomsky E. Plessier F. Mailhe M. P. Renno J. (2018). Cnidarian cell type diversity and regulation revealed by whole-organism single-cell RNA-Seq. *Cell* 173 1520–1534. 10.1016/j.cell.2018.05.019 29856957

[B47] Shannon P. Ramage D. Markie A. Amin N. (2003). Cytoscape: a software environment for integrated models of biomolecular interaction networks. *Genome Res.* 13 2498–2504. 10.1101/gr.1239303 14597658 PMC403769

[B48] Smith A. Avaron F. Guay D. Padhi B. K. Akimenko M. A. (2006). Inhibition of BMP signaling during zebrafish fin regeneration disrupts fin growth and scleroblasts differentiation and function. *Dev. Biol.* 299 438–454. 10.1016/j.ydbio.2006.08.016 16959242

[B49] Spanjaard B. Hu B. Mitic N. Olivares-Chauvet P. Janjuha S. Ninov N. (2018). Simultaneous lineage tracing and cell-type identification using CRISPR-Cas9-induced genetic scars. *Nat. Biotechnol.* 36 469–473. 10.1038/nbt.4124 29644996 PMC5942543

[B50] Srivastava M. Mazza-Curll K. L. van Wolfswinkel J. C. Reddien P. W. (2014). Whole-body acoel regeneration is controlled by Wnt and Bmp-Admp signaling. *Curr. Biol.* 24 1107–1113. 10.1016/j.cub.2014.03.042 24768051

[B51] Steiner A. B. Kim T. Cabot V. Hudspeth A. J. (2014). Dynamic gene expression by putative hair-cell progenitors during regeneration in the zebrafish lateral line. *Proc. Natl. Acad. Sci. U.S.A.* 111 E1393–E1401. 10.1073/pnas.1318692111 24706895 PMC3986164

[B52] Tanaka E. M. (2016). The molecular and cellular choreography of appendage regeneration. *Cell* 165 1598–1608. 10.1016/j.cell.2016.05.038 27315477

[B53] Tang Q. Iyer S. Lobbardi R. Moore J. C. Chen H. Lareau C. (2017). Dissecting hematopoietic and renal cell heterogeneity in adult zebrafish at single-cell resolution using RNA sequencing. *J. Exp. Med.* 214 2875–2887. 10.1084/jem.20170976 28878000 PMC5626406

[B54] Thisse C. Thisse B. (2008). High-resolution in situ hybridization to whole-mount zebrafish embryos. *Nat. Protoc.* 3 59–69. 10.1038/nprot.2007.514 18193022

[B55] Torraca V. Mostowy S. (2018). Zebrafish infection: from pathogenesis to cell biology. *Trends Cell Biol.* 28 143–156. 10.1016/j.tcb.2017.10.002 29173800 PMC5777827

[B56] Tosches M. A. Yamawaki T. M. Naumann R. K. Jacobi A. A. Tushev G. Laurent G. (2018). Evolution of pallium, hippocampus, and cortical cell types revealed by single-cell transcriptomics in reptiles. *Science* 360 881–888. 10.1126/science.aar4237 29724907

[B57] Vento-Tormo R. Efremova M. Botting R. A. Turco M. Y. Vento-Tormo M. Meyer K. B. (2018). Single-cell reconstruction of the early maternal-fetal interface in humans. *Nature* 563 347–353. 10.1038/s41586-018-0698-6 30429548 PMC7612850

[B58] Wagner D. E. Weinreb C. Collins Z. M. Briggs J. A. Megason S. G. Klein A. M. (2018). Single-cell mapping of gene expression landscapes and lineage in the zebrafish embryo. *Science* 360 981–987. 10.1126/science.aar4362 29700229 PMC6083445

[B59] Wang J. Sun H. Jiang M. Li J. Zhang P. Chen H. (2021). Tracing cell-type evolution by cross-species comparison of cell atlases. *Cell Rep.* 34:108803. 10.1016/j.celrep.2021.108803 33657376

[B60] Weng Q. Wang J. Wang J. He D. Cheng Z. Zhang F. (2019). Single-cell transcriptomics uncovers glial progenitor diversity and cell fate determinants during development and gliomagenesis. *Cell Stem Cell* 24 707–723. 10.1016/j.stem.2019.03.006 30982771 PMC6669001

[B61] Wolf F. A. Angerer P. Theis F. J. (2018). SCANPY: large-scale single-cell gene expression data analysis. *Genome Biol.* 19:15. 10.1186/s13059-017-1382-0 29409532 PMC5802054

[B62] Wu C. C. Kruse F. Vasudevarao M. D. Junker J. P. Zebrowski D. C. Fischer K. (2016). Spatially resolved genome-wide transcriptional profiling identifies bmp signaling as essential regulator of zebrafish cardiomyocyte regeneration. *Dev. Cell* 36 36–49. 10.1016/j.devcel.2015.12.010 26748692

[B63] Zheng W. Wang Z. Collins J. E. Andrews R. M. Stemple D. Gong Z. (2011). Comparative transcriptome analyses indicate molecular homology of zebrafish swimbladder and mammalian lung. *PLoS One* 6:e24019. 21887364 10.1371/journal.pone.0024019 PMC3162596

