# Supplementary figures and images for: Characterization of the Zebrafish Cell Landscape at Single-Cell Resolution
Source: Front Cell Dev Biol. 2021 Oct 1;9:743421. doi: 10.3389/fcell.2021.743421 (PMC8517238; doi:10.3389/fcell.2021.743421)

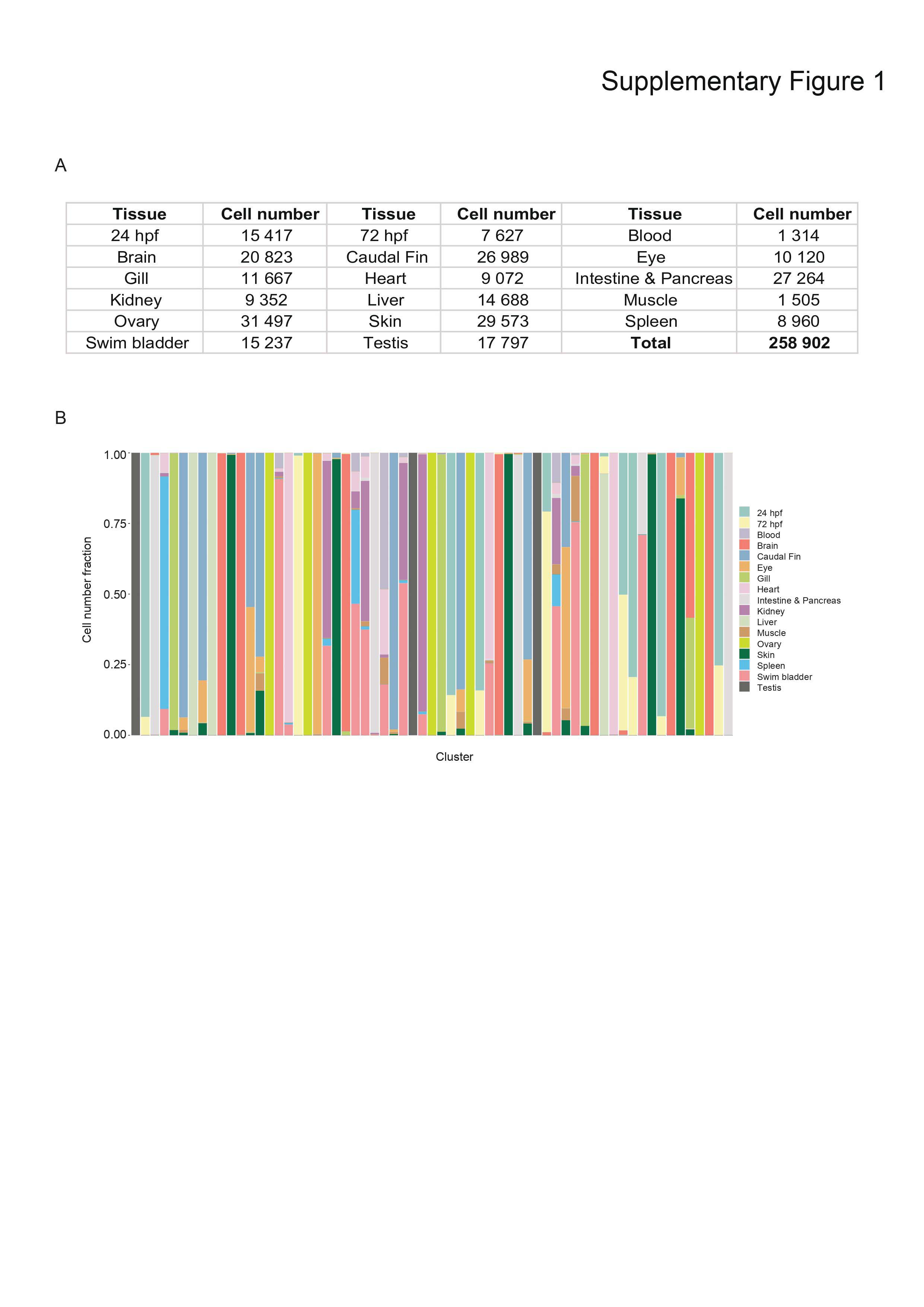

Supplement: Supplementary Figure 1 — Constructing a zebrafish cell landscape using microwell-seq. (A) Number of cells currently processed in zebrafish cell landscape. (B) The cluster contribution bar charts in zebrafish cell landscape. [file Image_1.JPEG]

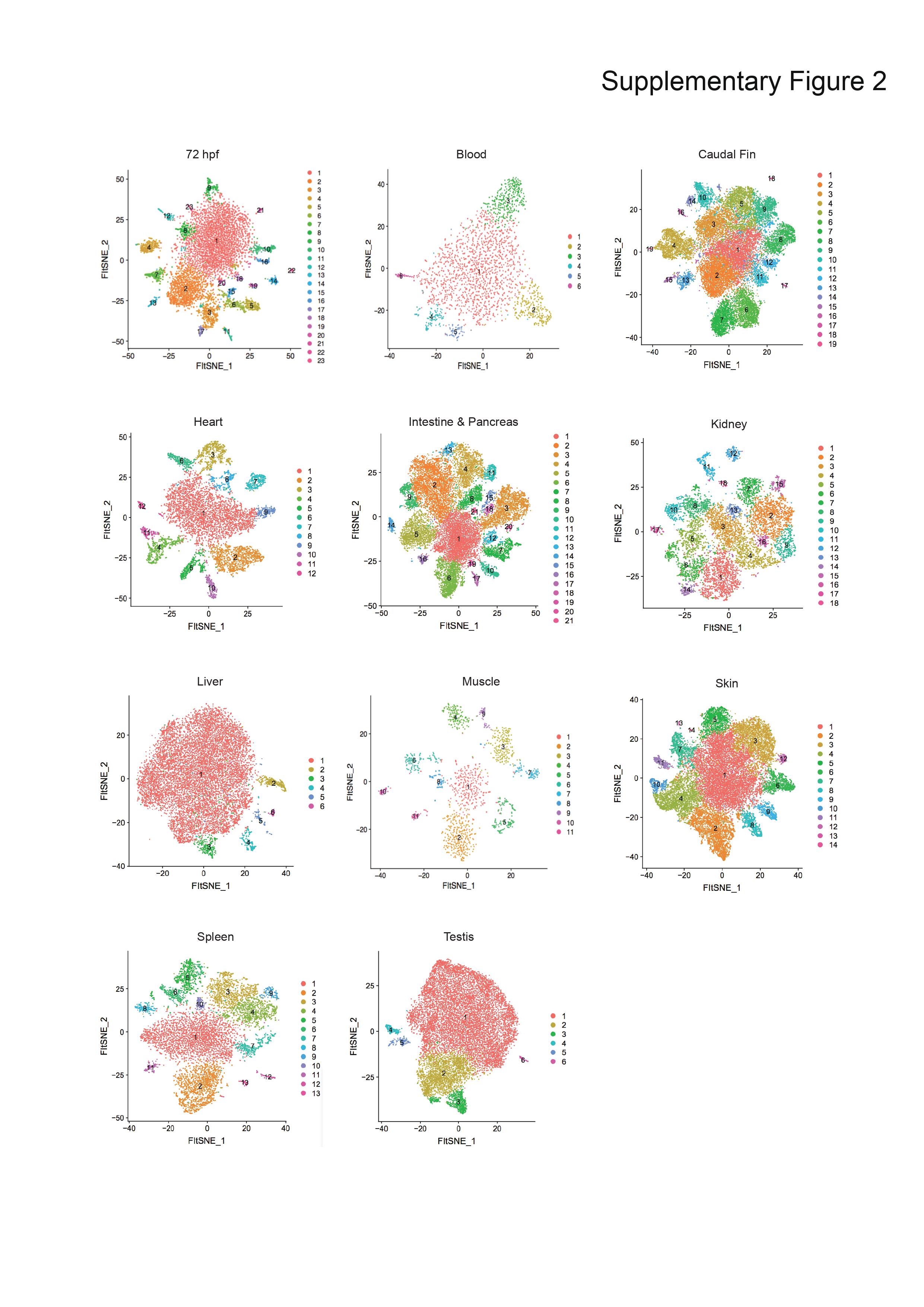

Supplement: Supplementary Figure 2 — Cellular heterogeneity in embryo and adult tissues. t-SNE map of zebrafish embryo (72 hpf), blood, caudal fin, heart, intestine, pancreas, kidney, liver, muscle, skin, spleen, testis single-cell data. Cells are colored by cell-type cluster. hpf, hours post-fertilization. [file Image_2.JPEG]

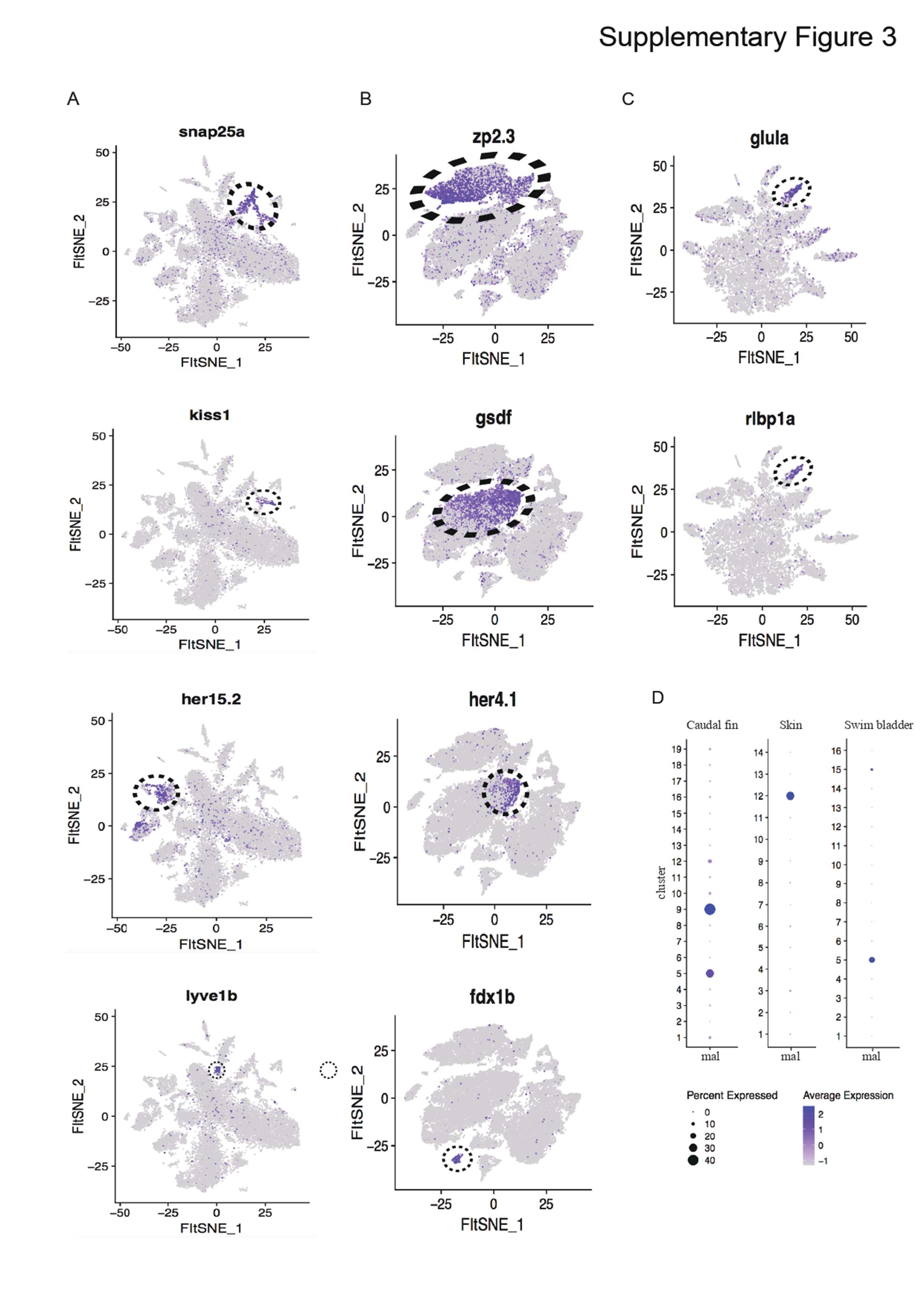

Supplement: Supplementary Figure 3 — Signatures of gene expression in adult tissues. (A) Feature plot showing representative gene expression in zebrafish brain. (B) Feature plot showing representative gene expression in zebrafish ovary. (C) Feature plot showing representative gene expression in zebrafish eye. (D) Dot plot showing the expression of mal in zebrafish caudal fin, skin, and swim bladder. [file Image_3.JPEG]

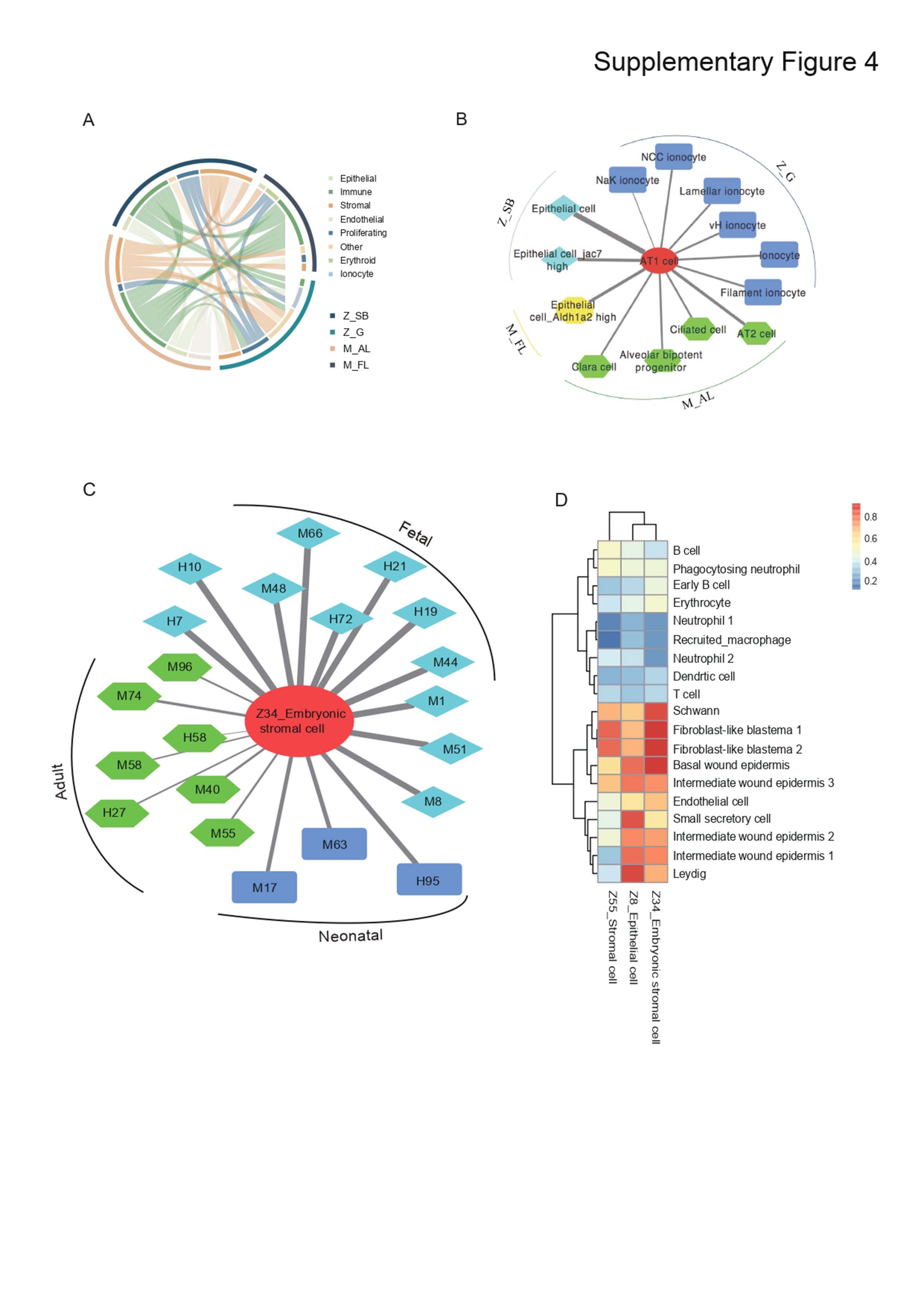

Supplement: Supplementary Figure 4 — Cross-species analysis of cell-type similarity. (A) Circos plot showing the similarity of cell types in zebrafish gill, swim bladder and mouse lung. Paired cell types with average AUROC scores greater than 0.8 are connected by lines. (B) A cell-type correlation network between lung AT1 cell and swim bladder epithelial cell, gill ionocyte and other lung epithelial cell. Thick lines indicate high correlation; thin lines indicate low correlation. Z_SB, zebrafish swim bladder; Z_G, zebrafish gill; M_AL, mouse adult lung; M_FL, mouse fetal lung. (C) A cell–cell correlation network between zebrafish embryonic stromal cells and human, mouse fetal, neonatal, adult stromal cells. Thick lines indicate high correlation; thin lines indicate low correlation. H, human; M, mouse; Z, zebrafish. (D) Correlation between zebrafish embryonic stromal cell, stromal cell, epithelial cell and axolotl limbs regenerative landscape. Red indicates high correlation; blue and yellow indicate low correlation. Z, zebrafish. [file Image_4.JPEG]

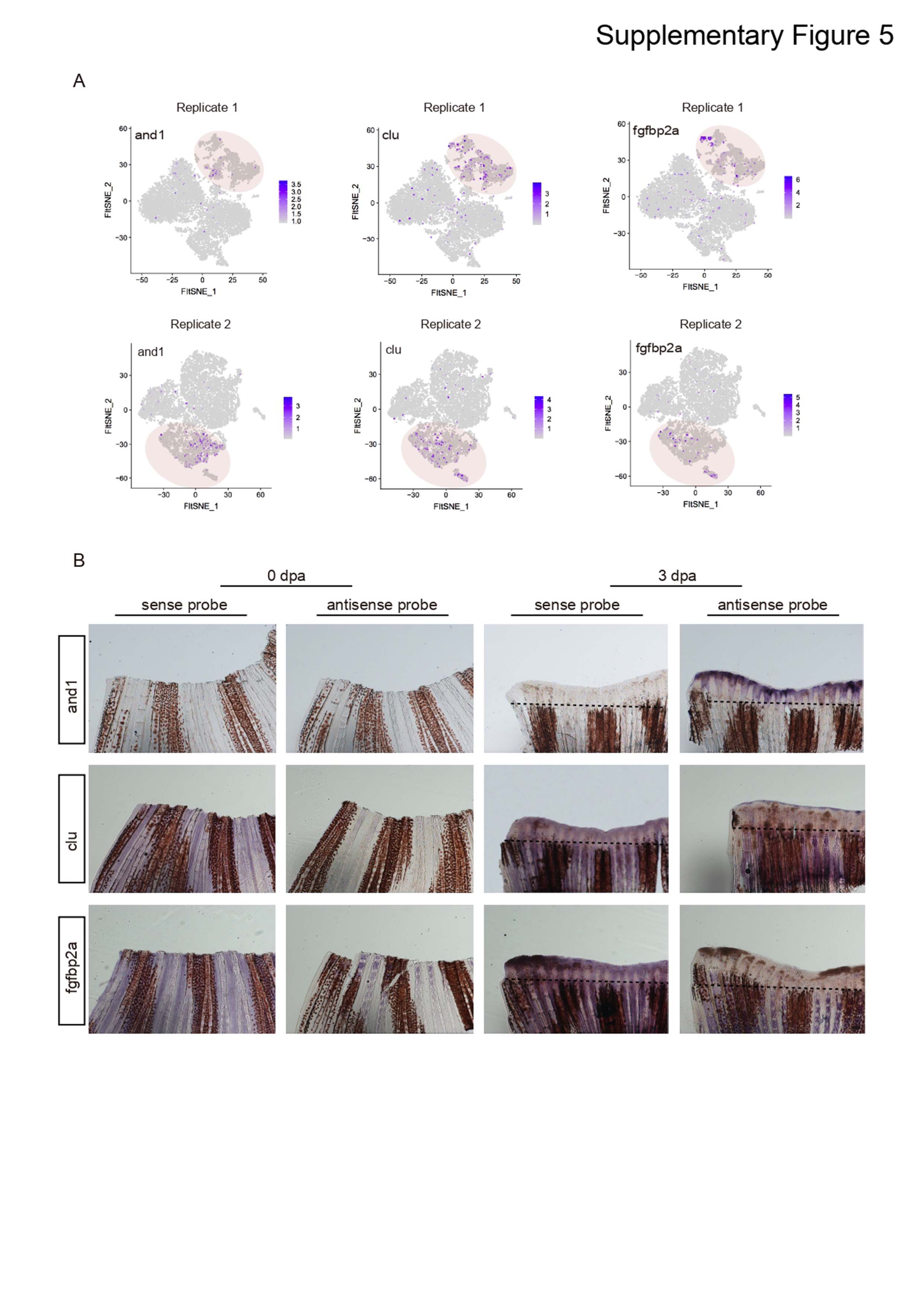

Supplement: Supplementary Figure 5 — Characteristic of blastema cells during caudal fin regeneration. (A) Feature plot showing high expression of and1, clu, and fgfbp2a in caudal fin regeneration single-cell dataset from Replicate 1 and Replicate 2. (B) Whole mount in situ hybridization against marker genes with sense and antisense probe in caudal fin at 0 and 3 dpa. Dashed lines indicate the amputation planes. n = 3 independent experiments. dpa, days post-amputation. scale bars, 200 μm. [file Image_5.JPEG]

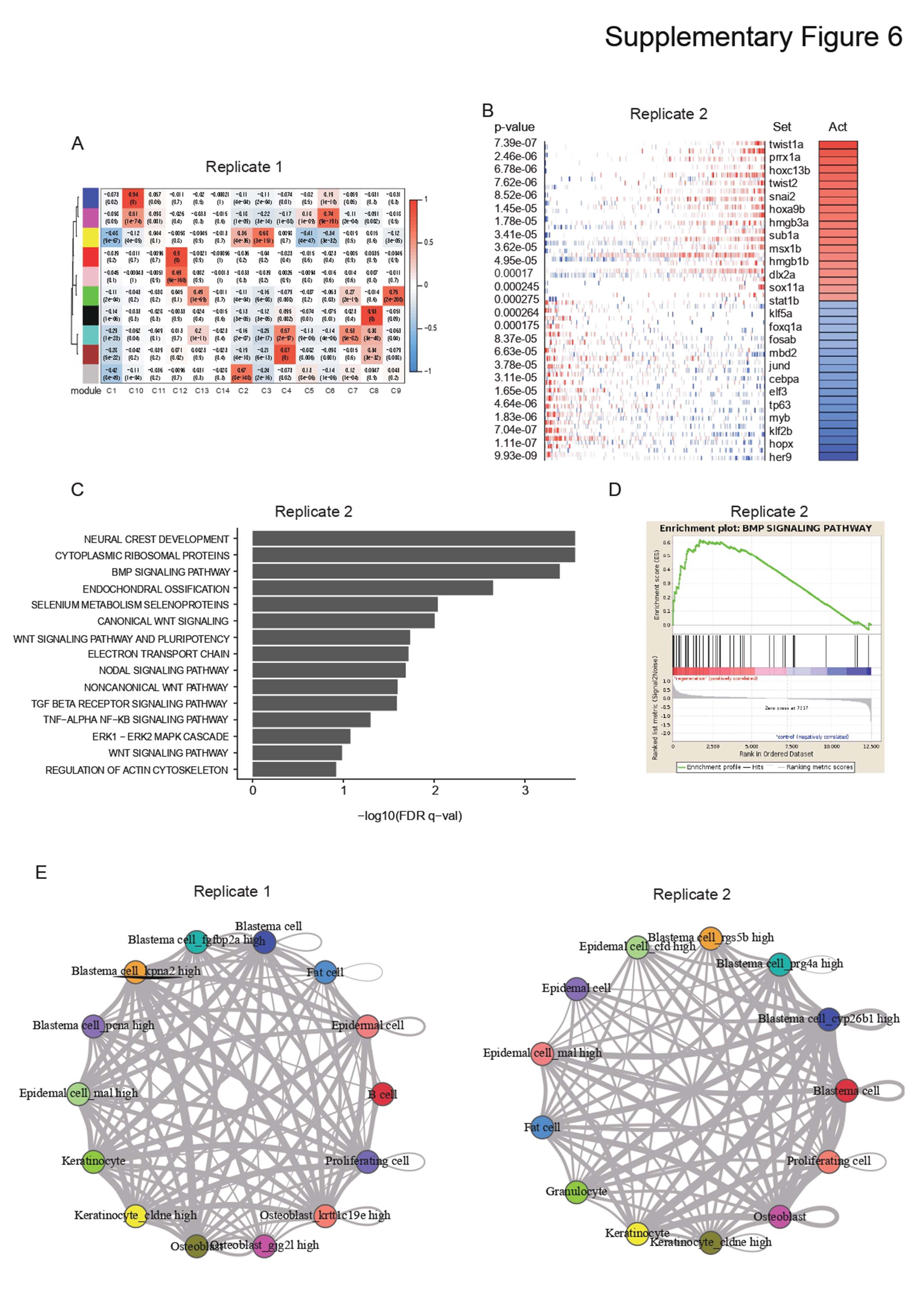

Supplement: Supplementary Figure 6 — Genetic regulation during tissue regeneration. (A) Heatmap showing correlation between caudal fin regeneration modules and cell types from Replicate 1. Red corresponds to a high correlation; blue and white correspond to low correlation. (B) Virtual inference of protein-activity by enriched regulon analysis in caudal fin Replicate 2. Red represents activated transcription factors; blue indicates repressed transcription factors. Act, activation. (C) Gene set enrichment analysis of caudal fin regeneration module in Replicate 2. (D) Gene set enrichment analysis between caudal fin regeneration module and non-regeneration module in Replicate 2. (E) Ligand and receptor analysis of caudal fin regeneration by CellPhoneDB. The colors represent cell types; line thickness indicates the degree of association between cell types. [file Image_6.JPEG]
